# TIPE3 represses head and neck squamous cell carcinoma progression via triggering PGAM5 mediated mitochondria dysfunction

**DOI:** 10.1038/s41419-023-05775-3

**Published:** 2023-04-06

**Authors:** Wei Chen, Xijuan Chen, Lixuan Wang, Rongchun Yang, Weilin Zhang, Siyuan Zhang, Juan Xia, Bin Cheng, Tong Wu, Xianyue Ren

**Affiliations:** 1 Hospital of Stomatology, Sun Yat-sen University, Guangzhou, Guangdong 510055 China; 2 Guangdong Provincial Key Laboratory of Stomatology, Guangzhou, Guangdong 510055 China; 3 Guanghua School of Stomatology, Sun Yat-sen University, Guangzhou, Guangdong 510055 China

**Keywords:** Head and neck cancer, Apoptosis, Prognostic markers

## Abstract

Mitochondria are essential organelles in balancing oxidative stress and cell death during cancer cell proliferation. Rapid tumor growth induces tremendous stress on mitochondria. The mammalian tumor necrosis factor-α-induced protein 8-likes (TIPEs) family plays critical roles in balancing cancer cell death and survival. Yet, the roles of TIPEs in HNSCC tumorigenesis and mitochondria stress maintenance is unclear. Based on an integrative analysis of public HNSCC datasets, we identified that the downregulation of TIPE3 via its promoter hypermethylation modification is the major event of TIPEs alterations during HNSCC tumorigenesis. Low expression levels of TIPE3 were correlated with high malignancy and poor clinical outcomes of HNSCC patients. Restoring TIPE3 represses HNSCC proliferation, migration, and invasion in vitro and in vivo, while silencing TIPE3 acted on an opposite way. Mechanistically, TIPE3 band to the PGAM5 and electron transport chain (ETC) complex. Restoring TIPE3 promoted PGAM5 recruiting BAX and dephosphorylating p-DRP1(Ser637), which triggered mitochondrial outer membrane permeabilization and fragmentation. Ultimately, TIPE3 induced ETC damage and oxygen consumption rate decrease, ROS accumulation, mitochondrial membrane potential depolarization, and cell apoptosis. Collectively, our work reveals that TIPE3 plays critical role in maintaining mitochondrial stress and cancer cell progression in HNSCC, which might be a potential therapeutic target for HNSCC patients.

## Introduction

Head and neck squamous cell carcinoma (HNSCC) is the 7th most common human cancer with high heterogeneity and mortality in the worldwide [1, 2]. Although the disease management has been improved greatly during the past decades, the 5-year survival rate has not improved obviously and is still below 60%. Only a few patients benefit from the emerging strategies, such as anti-PD1/PD-L1 immunotherapy [2, 3]. Exploring the mechanism of HNSCC progression and developing new therapeutic target are still the primary missions for current study.

Sustaining proliferation and resisting apoptosis are the hallmark traits of cancers [4, 5]. As central hubs for energy metabolism, redox maintenance, and macromolecular synthesis, mitochondria control extensive signaling networks to balance cellular proliferation and programmed cell death [6–8]. Mitochondria are double-membrane dynamic organelles with constant fission, fusion, and mitophagy to maintain their morphology and functions. The two proteolipid membranes of mitochondria establish at least five compartments, including MOM (mitochondrial outer membranes), MIS (intermembrane space), MIM (mitochondrial inner membranes), cristae, and matrix. Cristae is the primary site of electron transport by the respiratory machinery afterward ATP synthesis and reactive oxygen species (ROS) generation. Following diverse stresses, the breakdown of the mitochondrial dynamic equilibrium caused mitochondrial dysfunction. An accumulation of dysfunctional mitochondria is harmful to cells, potentially via increased ROS and mis-localized mitochondrial DNAs that lead to oxidative stress, increased mitochondrial outer membrane permeabilization (MOMP) or amplified apoptosis [9]. As such, considerable interest has focused on targeting of mitochondria to manipulate cell death in cancer [10, 11]. Understanding the mechanism of HNSCC cancer cells maintain the mitochondrial stress hemostasis is of great meaningful.

The mammalian tumor necrosis factor-α-induced protein 8 (TNFAIP8)-like (TIPEs) family sharing a homologous death effector domain (DED) consists of four proteins, namely: TNFAIP8 (TIPE), TNFAIP8L1 (TIPE1), TNFAIP8L2 (TIPE2), and TNFAIP8L3 (TIPE3) [12–14]. High sequence and structural similarities are shared within the family, including a highly conserved TIPE2 homology (TH) domain with a hydrophobic cavity which could capture and shuttle the lipid second messengers like phosphatidylinositol 4,5-bisphosphate (PtdIns(4,5)P2) and phosphatidylinositol 3,4,5-trisphosphate (PtdIns(3,4,5)P3). Nevertheless, the function of TIPE family varied greatly in different cancer types. In majority cancers, TIPE and TIPE3 were realized as anti-apoptotic and pro-survival proteins, while TIPE1 and TIPE2 were considered to be potential pro-apoptotic and anti-tumor molecules. TIPEs play vital roles in cellular destiny determination, yet, little is known about their roles in HNSCC progression and mitochondrial hemostasis maintenance.

In the present study, we comprehensively assessed the alterations of TIPEs in HNSCC patients. Through in vitro and in vivo approaches, we demonstrated that the downregulation of TIPE3 might be responsible for the reconstruction of mitochondria stress hemostasis and help cancer cells evading from cell death in HNSCC, which might uncover a novel molecular mechanism regulating mitochondrial stress hemostasis and provide a novel insight into clinical therapy strategy development for HNSCC patients.

## Methods

### Clinical cohorts and bioinformatic analysis

The gene expression profiles from TCGA-HNSC (normal = 44, HNSCC = 520; IlluminaHi-Seq RNASeq), GSE37991 (normal = 40, HNSCC = 40; Illumina HumanRef-8 v3.0 expression beadchip), GSE56142 (normal = 12, HNSCC = 12; Illumina HumanHT-12 V4.0 expression beadchip), GSE25099 (normal = 22, HNSCC = 57; Affymetrix Human Exon 1.0 ST Array), GSE30784 (normal = 45, dysplasia = 17, HNSCC = 167; Affymetrix Human Genome U133 Plus 2.0 Array) were enrolled to determine the mRNA alterations of TIPEs in normal tissues and HNSCC tissues. The Infinium Human Methylation 450 K BeadChip microarray from TCGA-HNSC (normal = 20, HNSCC = 520), GSE75537 (normal = 54, HNSCC = 54), GSE123781 (normal = 18, HNSCC = 15), GSE87053 (normal = 10, HNSCC = 11) were enrolled to determine the methylation alterations of TIPE3 in normal tissues and HNSCC tissues.

Kaplan–Meier survival analysis and univariate COX analysis were used to identify survival-related TIPEs. The cutoff value for high (TIPE3^high^) and low (TIPE3^low^) expression levels was determined using Receiver operating characteristic (ROC) curve analysis. Multivariate Cox regression analysis with the backward stepwise method was performed to determine the independent prognostic factors. The cBioPortal for Cancer Genomics (https://www.cbioportal.org) was used to determine the correlation between TIPE3 promoter methylation levels and mRNA levels. Pearson correlation analysis was performed to evaluate the correlation between the methylation levels of TIPE3 CG probes and mRNA levels. GSEA was performed to assess the different enrichment pathways of TIPE3 between normal tissues and HNSCC tissues. The ssGSEA algorithm of Gene Set Variation Analysis (GSVA) was applied to calculate the enrichment scores of pathways in HNSCC patients with TIPE3^high^ and TIPE3^low^.

### Cell culture

Human oral keratinocytes cell line (HOK) was purchased from ScienCell Research Laboratories, inc. (CA, USA). Human HNSCC cell lines (HSC2, HSC3, HSC4, UM1, Cal33, HN6) were maintained in our laboratory (Guangzhou, China) [15]. HSC3 and Cal33 were grown in Dulbecco’s modified Eagle medium (DMEM; Invitrogen, Thermo Fisher Scientific, CA, USA) containing 10% fetal bovine serum (FBS; Front/BOVOGEN, New Zealand). UM1 and HN6 were grown in DMEM/F12 medium (Invitrogen, Thermo Fisher Scientific, CA, USA) with 10% FBS. HSC2 and HSC4 were grown in Minimum Essential Medium (Invitrogen, Thermo Fisher Scientific, CA, USA) with 10% FBS. All cells were cultured at 37 °C in a humidified incubator at 5% CO_2_.

### 5-Aza-2′-deoxycytidine (decitabine, DAC) and Mdivi-1 treatment

For the methyltransferase inhibitor 5-Aza-2′-deoxycytidine (A3656, Sigma-Aldrich, USA) treatment [16], 1.5 × 10^5^ cells were seeded on 60 mm culture dishes. After growing for 24 h, cells were treated with DMSO or 5-Aza (2.5 μM) by replacing the drug every 24 h for 72 h. After that, the cells were collected for DNA or RNA extraction. For the Mdivi-1 treatment, 1.5 × 10^5^ TIPE3 or vector overexpression cells were seeded on 60 mm culture dishes. After growing for 24 h, cells were treated with DMSO or 1 μM Mdivi-1 for 24 h. After that, the cells were collected for subsequent analysis.

### DNA extraction and bisulfite pyrosequencing analysis (BSP)

According to the manufacturer’s instructions, cells genomic DNA was extracted using a TIANamp Genomic DNA Kit (China). BSP was conducted by GeneTech Co., Ltd. (Shanghai, China) [17]. Briefly, DNA bisulfite modification was conducted using an EpiTect Bisulfite Kit (Qiagen). Bisulfite pyrosequencing primers were designed using PyroMark Assay Design Software 2.0. Sequencing reaction and methylation level quantification were performed by the PyroMark Q96 ID System and software (Qiagen). The primer sequences for PCR and sequencing are shown in Supplementary Table 1.

### RNA extraction and real-time quantitative PCR (RT qPCR)

Total RNA from cells was extracted using RNA-Quick Purification Kit (YISHAN Biotechnology Co., Shanghai, China) following the manufacturer’s instruction. The cDNA was synthesized using GoScript^TM^ Reverse Transcription Mix (Promega, USA) as previously described. Then, SYBR Green-based (Invitrogen) qPCR analysis was run using the CFX96 Touch™ sequence detection system (Bio-Rad, USA) [18]. Comparative threshold cycle (2-ΔΔCT) equation was used to calculate the relative expression levels. GAPDH was considered as an endogenous control. The primers are presented in Supplementary Table 1.

### Plasmid transfection and RNA interference

The pSin-EF2-TIPE3-HA (NM_001311175.1), pSin-EF2-trTIPE3-HA, and pSin-EF2-NT-HA plasmids were obtained from Long Bioscience (China). All the plasmids were confirmed by DNA sequencing (Tsingke Biotechnology Co., Guangzhou, China) before use. The pSin-EF2-puro-Vector plasmid was used as negtive control.

HNSCC cells stably overexpressed TIPE3 were constructed as we previously reported [17, 19]. Briefly, the pSin-EF2-TIPE3-HA or empty vector, as well as the lentivirus packaging plasmids psPAX2 and pMD2.G, were co-transfected into 293FT cells using the calcium phosphate method. After transfection, lentivirus particles were gained and applied to infect Cal33 and HN6 cells. The stably TIPE3 overexpression cells were selected by puromycin (Sigma, USA) and confirmed with qPCR and western blotting.

Short interference RNAs (siRNAs) provided by RiboBio Co., Ltd. (Guangzhou, China) or plasmids were transfected into cells using Lipofectamine 3000 reagent (Invitrogen, USA) according to the manufacturer’s instruction. After transfection for 24–36 h, cells were harvested for the subsequent experiments. RT-qPCR was performed to examine the knocking down efficiencies. The si-NC was set as negative control. The sequences of siTIPE3s are presented in Supplementary Table 1.

### Live-cell imaging, cell counting Kit-8 (CCK8), and colony formation assays

For real-time live-cell proliferation evaluation, the IncuCyte live-cell imaging system (Essen Biosciences, USA) was utilized. Cells were plated in a 96-well plate and imaged and quantified by IncuCyte®software.

For cell viability assessment, the CCK8 (DOJINDO, Japan) was applied. About 1 × 10^3^ cells per well were seeded in 96-well plates. After incubating for the indicated times, cells were incubated with the mixture of 10 μl CCK8 and 100 μl FBS-free medium for another 2 h before detection. The cell viability was recorded at 560 nm using a spectrophotometric plate reader (Biotek, USA).

For the colony formation assay, 500 cells per well were plated in 6-well plates. After incubating for the indicated times, the colonies were fixed with methyl alcohol, stained with 0.5% crystal violet, and then counted under an inverted microscope.

### Transwell migration and invasion assays

Transwell chamber with 8 μm pores in the membrane (BD Falcon, USA) coated with or without Matrigel were applied to detect the migration or invasion ability of cells, respectively. Briefly, 8 × 10^4^ Cal33 or 3 × 10^4^ HN6 cells suspended in serum-free medium were added into the upper chambers, while 20% or 10% FBS was placed into the lower chambers, respectively. After climbing for 24 h, the migrated or invaded cells were fixed with methyl alcohol, stained with 0.5% crystal violet, and counted under an inverted microscope (ZEISS, Germany).

### Western blotting (WB) assay

Briefly, total proteins of cells were lysed using RIPA buffer (Beyotime Biotechnology, China) supplemented with protease and phosphatase inhibitors. lysates were loaded onto SDS-PAGE gel for separation and transferred to PVDF membrane (Millipore, USA). Then, PVDF membranes were blocked with 5% milk and incubated with primary antibodies at 4 °C overnight, followed by incubating with species-matched secondary antibody. The signal was visualized using an enhanced chemiluminescence detection system (Millipore). The antibodies used are shown in Supplementary Table 2.

### Co-immunoprecipitation (Co-IP) and mass spectrometry (MS)

Briefly, total proteins of cells were lysed using IP lysis buffer (Thermo Fisher Scientific, USA) with protease and phosphatase inhibitors. Primary antibodies were incubated with the cell lysates overnight at 4 °C. Then, protein A/G Sepharose beads (Thermo Fisher Scientific, USA) were added into the immune-complexes for recovery. After that, the immune-complexes were washed and harvested for mass spectrometry analysis (Huijun Biotechnology, Guangzhou, China) or western blotting. Metascape (http://metascape.org/gp/index.html) was performed to analyze the enrichment pathway of interactors [20]. Western blotting was used to confirm the proteins of interest. Anti-IgG antibody was used as a negative control. The antibodies used for IP assays are shown in Supplementary Table 2.

### Immunofluorescence assay

Cells were harvested and fixed with methyl alcohol. After permeabilized in phosphate-buffered saline (PBS) with 0.5% Triton X-100, cells were incubated with the primary antibodies at 4 °C overnight. Then, cells were stained with species-matched secondary antibodies. Nuclei were stained with DAPI (Sigma-Aldrich). The slides were viewed using confocal laser-scanning microscopes (LSM 780, ZEISS; FV3000, Olympus). The antibodies used were shown in Supplementary Table 2.

### Transmission electronic microscopy (TEM)

TEM was applied to observe the morphology alteration of mitochondria. Briefly, cells were fixed with 2.5% glutaraldehyde, post-fixed with 1% osmium tetroxide/phosphate buffer (OsO4), and dehydrated with gradient ethanol alcohol. Then, cells were penetrated and embed with resin. After polymerization and ultrathin section, the sections were stained with 2% uranium acetate saturated alcohol solution and 2.6% Lead citrate. Finally, the cuprum grids are observed under TEM (HT7800, Hitachi).

### Mitochondria mass analysis

MitoTracker™ Red CMXRos Kit (Thermo) was used to staining the mitochondria. Cells were fixed with 4% formaldehyde, permeabilized with 0.2% Triton® X-100 and incubated with MitoTracker® probe at a final concentration of 100 nM at 37 °C for 15 min. Next, cells were replaced staining solution with FBS-free culture media. Finally, cells were observed using confocal laser-scanning microscopes (LSM 780, ZEISS; FV3000, Olympus).

### Flow cytometry analyses

For apoptosis assay, the Annexin V-FITC/PI Apoptosis Detection Kit (Telenbiotech, China) was used. Briefly, cells were cultured with FBS-free culture medium. After 24 h starvation, cells were collected and stained with FITC and PI for 15 min at room temperature. The apoptotic cells were measured by flow cytometry (Cytoflex, Beckman).

For reactive oxygen species (ROS) analysis, Reactive Oxygen Species Assay Kit (Beyotime Biotechnology, China) was utilized to assess the total ROS levels of cells, while the MitoSOX^TM^ Red Kit (Invitrogen^TM^, Thermo Fisher Scientific, USA) was used to assess the mitochondrial ROS levels. After staining with ROS probes, flow cytometry was used to detect the production of ROS in cytoplasm and mitochondria.

The mitochondrial membrane potential (MMP) was determined by Mitochondrial membrane potential assay kit with JC-1 (Beyotime Biotechnology, China). When the MMP decrease, J-aggregates form (red fluorescence) released from mitochondria and turned to monomer form (green fluorescence) in the cytoplasm. Cells were collected and dyed with the JC-1 for 20 min at 37 °C. Next, the flow cytometer was performed to determine the intensities of the red and green fluorescences. The MMP was calculated as the ratio of red signals versus the green signals.

### Mitochondrial function analysis

The XF Cell Mito Stress Test Kit (Agilent) was used to measure oxygen consumption rate (OCR) according to the manufacturer’s protocol by Seahorse XF96 Extracellular Flux Analyzer (Agilent, USA) [21]. Briefly, CAL33 cells or HN6 cells were plated onto the Seahorse 96-well plates and incubated overnight. The cell media were replaced with the assay medium (XF Base Medium supplied with 1 mM pyruvate, 2 mM glutamine, and 10 mM glucose, and adjusted pH to 7.4 with 0.1 N NaOH) before testing. Cells were sequentially treated with Oligomycin (Oligo, a Fo-ATPase inhibitor of Complex V, could significantly reduce mitochondrial respiration; 1 μM), uncoupler carbonyl cyanide-ptrifluoromethoxyphenylhydrazone (FCCP; 2 μM), rotenone (Rot) and antimycin A (0.5 μM). The oxygen consumption rates were recorded by the XF96 Extracellular Flux Analyzer and normalized to cell protein levels in each well.

### In vivo xenograft tumor models

All animal research procedures were carried on according to the detailed rules of the Institutional Animal Care and Use Committee of Sun Yat-sen University. Twelve female BALB/c nude mice (4–6 weeks old) were randomly divided into 2 groups (six individuals per group). HN6 cells with vector or TIPE3 overexpression (1 × 10^6^ cells) were subcutaneously injected into the right armpit region. The primary tumor sizes were recorded every 3 days. Tumor volumes were estimated as our previously reported. After 2 weeks’ growth, the mice were sacrificed for primary tumors and axillary lymph nodes. The tumors and lymph nodes were weighed, paraffin-embedded, and cut into 5 mm tissue slices for H&E and IHC staining.

### Immunochemistry (IHC) analysis

IHC was performed on xenograft mice tissues [19]. Briefly, xylene was used to deparaffinize and alcohol with gradient concentration was applied to rehydrate; the endogenous peroxidase activity was blocked by 3% H_2_O_2_. Then, slices were subjected to citrate-mediated high-temperature antigen retrieval. After that, the nonspecific binding was subsequently blocked, and the slices were incubated with the primary antibodies and species-matched secondary antibodies. Sections were scanned and scored using an Apreio AT2 digital whole slide scanner (Leica, Wetzlar, Germany). Antibodies used are shown in Supplementary Table 2.

### Statistics

R (version 4.0.3), SPSS 25.0 (SPSS Inc., Chicago, IL, USA), and GraphPad Prism 8.0 (GraphPad) were used to process and analyze the data. Data presented as the mean ± SD were extracted from at least three independent experiments. Based on two-sided tests, *P* value <0.05 was considered as statistically significant. Continuous variables and categorical variables were compared using Student’s t-test, or chi-square and Fisher’s exact tests.

## Results

### TIPE3 downregulation is related to promoter hypermethylation and poor prognosis of HNSCC patients

**Fig. 1:**
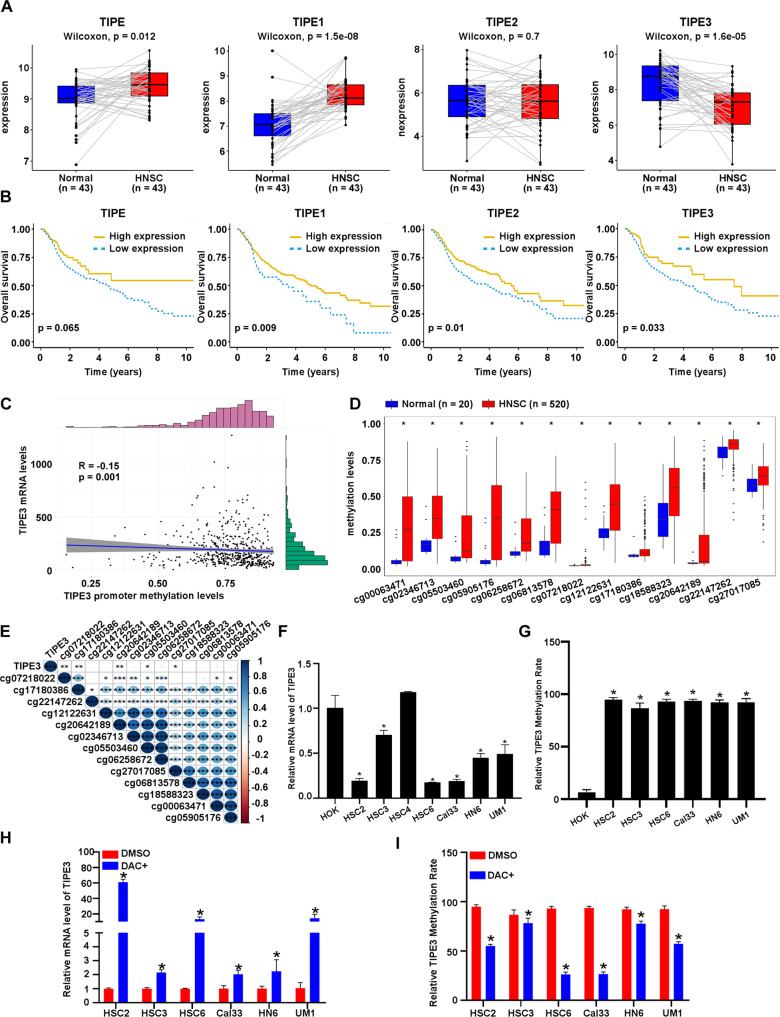
TIPE3 hypermethylation and downregulation is associated with poor survival of HNSCC patients. **A** The mRNA levels of TIPEs in paired adjacent normal tissues and HNSC tissues based on the TCGA database. **B** Kaplan–Meier curves of Overall survival according to the mRNA levels of TIPEs in HNSCC patients from the TCGA database. **C** Pearson correlation analysis of TIPE3 mRNA levels and promoter methylation levels performed using cBioPartal based on the TCGA database. **D** Methylation levels of CG probes of TIPE3 in normal tissues and HNSC tissues based on the TCGA database. **E** Pearson correlation analysis of TIPE3 mRNA levels and CG probes methylation levels of TIPE3 based on the TCGA database. **F** RT-qPCR analysis of TIPE3 mRNA levels in normal epithelial cell line HOK and HNSCC cell lines. **G** BSP analysis of cg20642189 methylation levels in normal epithelial cell line HOK and HNSCC cell lines. **H**, **I** TIPE3 methylation levels measured via BSP analysis (**H**) and relative TIPE3 mRNA levels measured via RT-qPCR (**I**) with DMSO or with DAC (DAC+, 2.5 μM) treatment in HOK and HNSCC cell lines. Mean ± s.d.; *P < 0.05; Student’s t-tests.

We examined the mRNA levels of TIPEs in HNSCC tumors and normal epithelia tissues in TCGA-HNSC and 4 GEO datasets (GSE37991, GSE56142, GSE25099, GSE30784). As the results showed that, in comparation to the adjacent or non-adjacent normal epithelia tissues, only the mRNA levels of TIPE3 were consistently downregulated in HNSCC tissues in all the datasets (Fig. 1A, Supplementary Fig. 1A–D). Patients with late-stage (TNM III-IV) or with lymph node metastasis patients had lower TIPE3 mRNA levels than those with early-stage (TNM I-II) or without lymph node metastasis ones, respectively (Supplementary Fig. 2A, B). Then, Kaplan–Meier survival analysis was performed and exhibited that patients with low TIPE3 mRNA levels had poorer overall survival (OS), progression-free survival (PFS), and recurrence-free survival (RFS) than those with high levels (Fig. 1B, Supplementary Fig. 3A, B). Univariate COX survival analysis confirmed that low TIPE3 mRNA level was associated with high risk of death (Supplementary Fig. 4A). Multivariate Cox regression analysis revealed that TIPE3 was an independent prognostic indicator for OS in HNSCC patients (Supplementary Fig. 4B). Thus, these findings indicated that TIPE3 downregulation was a high-risk event in HNSCC patients.

To determine the mechanism of TIPE3 downregulation in HNSCC, we analyzed the relationship between the mRNA levels and the promoter methylation levels of TIPE3 using cBioportal online tool, which indicated an inverse correlation manner between those two parameters (Fig. 1C). Then, the methylation alterations of 28 CG probes in TIPE3 genome between HNSCC tumor and normal tissues were examined. Totally, 43% (12/28) CG probes had higher methylation levels in the tumor tissues than those in the normal tissues, most of which were also identified in GSE75537, GSE123781, and GSE87053 cohorts (Fig. 1D, Supplementary Fig. 5A–C). Pearson correlation analysis demonstrated that the methylation levels of cg07218022, cg17180386, cg20642189, cg05503460, and cg27017085 probes were negatively correlated with TIPE3 mRNA levels (Fig. 1E). UCSC genome browser displayed that cg07218022, cg17180386, cg20642189, and cg05503460 were located in the CpG islands with high H3K4me1, H3K4me3, and H3K27Ac enrichment (the histone modifications present at active promoters) [17] (Supplementary Fig. 5D).

Next, we assessed TIPE3 mRNA levels using qPCR and its promoter methylation levels by bisulfite pyrosequencing in normal HOK and HNSCC (HSC2, HSC3, HSC6, cal33, HN6, and UM1) cell lines. In contrast to HOK, all the HNSCC cell lines had lower TIPE3 mRNA levels, except HSC4 (Fig. 1F). The higher methylation levels of TIPE3 were also identified in those HNSCC cell lines than HOK cells (Fig. 1G). When treated HNSCC cells with demethylation drug (DAC, 2.5 μM), the methylation levels of TIPE3 were decreased and the mRNA levels of TIPE3 were increased substantially (Fig. 1H, I, Supplementary Fig. 6). Collectively, those results demonstrated that the downregulation of TIPE3 expression was correlated with its promoter hypermethylation in HNSCC, which might be the mainstay event of TIPEs family during HNSCC progression.

### TIPE3 suppresses cell viability, migration, and invasion of HNSCC cells

**Fig. 2:**
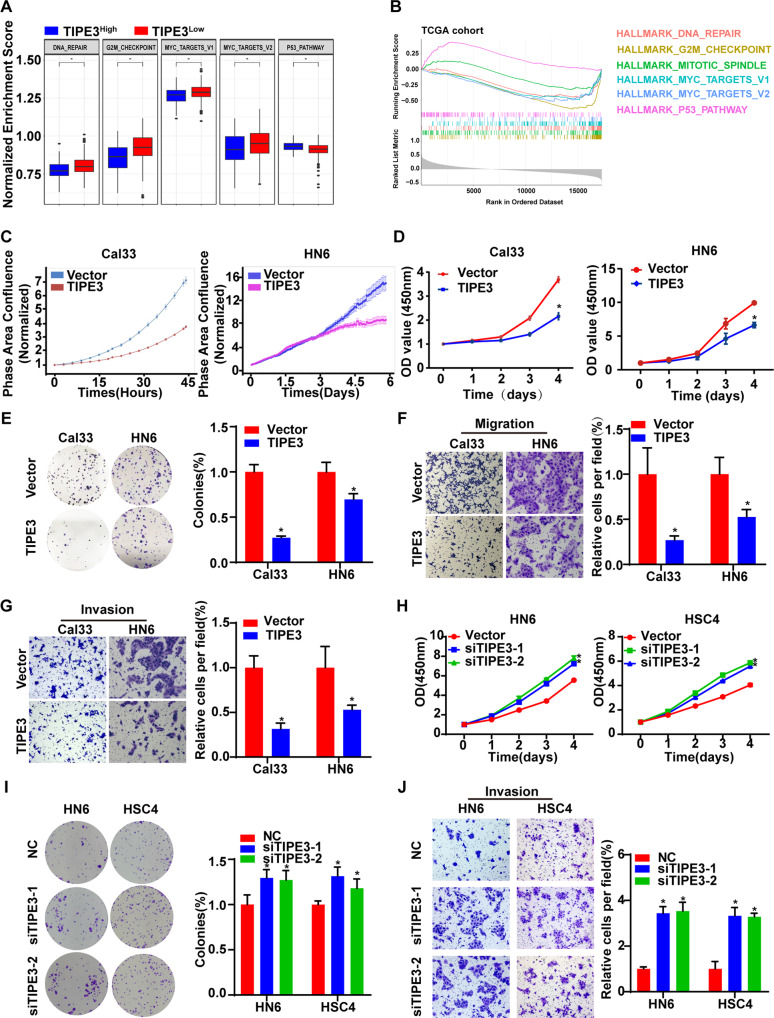
TIPE3 represses cell viability and migration in HNSCC cells. **A** The alterations of oncogenic signaling pathways between TIPE3^high^ and TIPE3^low^ HNSCC patients in TCGA HNSC dataset calculated using ssGSEA algorithm. **B** The oncogenic signaling pathways enrichment in genes correlated with TIPE3 expressions in HNSCC patients calculated using GSEA algorithm. **C**–**G** Cal33 and HN6 cells that stably overexpressed the vector or TIPE3 were used to examine the functions of restoring TIPE3 in HNSCC cells. The live-cell analysis of cell proliferation (**C**). CCK8 analysis of cell viability (**D**). Colony formation assay of cell colony forming ability (**E**). Transwell assay without or with matrigel was performed to detect the migration (**F**) or invasion (**G**) ability. **H**–**J** The si-NC or siTIPE3s transiently transferred into HN6 and HSC4 cells were used to examine the functions of silencing TIPE3 in HNSCC cells. CCK8 analysis of cell viability (**H**). Colony formation assay of cell colony forming ability (**I**). Transwell assay with matrigel was performed to detect the cellular invasion ability (**J**). Mean ± s.d.; *P < 0.05; Student’s t-tests.

To determine the biological functions of TIPE3 in HNSCC, firstly, we contrasted the differential expressions of oncogenic signaling pathways between TIPE3^low^ and TIPE3^high^ patients using ssGSEA algorithm. In comparation with TIPE3^low^ tumors, TIPE3^high^ tumors exhibited lower expressions of oncogenic pathways (DNA repair, G2M checkpoint, MYC targets scores) and higher expressions of tumor suppression pathways (P53 pathway) (Fig. 2A). Besides, the expressions of genes correlated with TIPE3 levels were also negatively enriched in G2M checkpoint, mitotic spindle, and MYC targets, and positively enriched in P53 pathway (Fig. 2B).

Then, we constructed TIPE3 stably overexpressed HNSCC cells using Cal33, HN6, and UM1 cells, and TIPE3 transiently silenced cells using HN6 and HSC4 cells. The transfection efficiencies were detected by qPCR and western blotting assays (Supplementary Fig. 7A, B). Live-cell analysis system observed that TIPE3 overexpression cells exhibited slower proliferation rates than vector cells (Fig. 2C, Supplementary Fig. 8A). CCK8 assay confirmed that overexpressing TIPE3 decreased cell viability (Fig. 2D, Supplementary Fig. 8B). Colony formation assay showed that single cancer cell survival and proliferation abilities were attenuated by restoring TIPE3 (Fig. 2E). Furthermore, the migration and invasion abilities of HNSCC cells were inhibited in TIPE3 overexpression cells (Fig. 2F, G). Moreover, silencing TIPE3 increased cell viability, colony formation, and invasion abilities (Fig. 2H–J). Hence, those data demonstrated that TIPE3 acted as a tumor suppressor gene in HNSCC.

### TIPE3 correlates with mitochondria stress in HNSCC cells

**Fig. 3:**
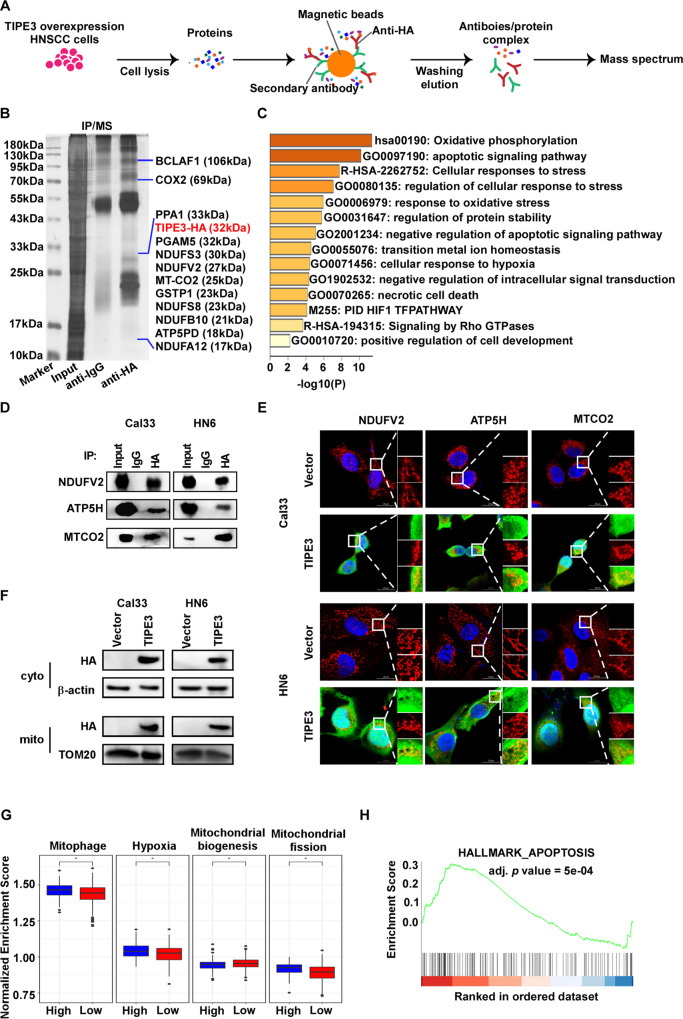
TIPE3 is co-localized to mitochondria and correlates with mitochondria stress. **A** Co-IP/MS was performed to identify the interactors of TIPE3. **B** Silver stained SDS-PAGE gel of proteins immunoprecipitated from Cal33 cells extract by HA. **C** Functional enrichment analysis of the interactors using Metascape. **D** Co-IP/WB was performed to clarify the interactions of NDUFV2, MTCO2, ATP5H with TIPE3-HA. **E** Immunofluorescence images of co-localization of TIPE3-HA (green) and NDUFV2 (red), ATP5H (red) or MTCO2 (red) expressions in Cal33 and HN6 cells with vector or TIPE3 stably overexpression. **F** WB was performed to examine the expressions of TIPE3-HA in cytoplasm and mitochondria. The expressions of GAPDH and TOM20 were considered as endogenous controls of cytoplasm and mitochondria proteins, respectively. **G** The ssGSEA analysis was performed to calculate the enrichment scores of mitophage, hypoxia, mitochondrial biogenesis, and mitochondrial fission of HNSC patients with TIPE3^high^ and TIPE3^low^ expressions in TCGA-HNSC cohort. **H** GSEA algorithm was used to evaluate enrichment of genes correlated with TIPE3 expressions based on TCGA-HNSC dataset. Mean ± s.d.; *P < 0.05; Student’s t-tests.

To illustrate the potential tumor suppression mechanism of TIPE3 in HNSCC, we performed co-IP/MS to identify the proteins interacted with TIPE3 in Cal33 cells (Fig. 3A). A total of 168 proteins were identified to be pulled down by TIPE3 protein (Supplementary Table 3). Notably, several electron transport chain (ETC) complex proteins located in the cristae of MIM (Complex I: NDUFA12, NDUFB10, NDUFS3, NDUFS8 and NDUFV2, Complex IV: MT-CO2, Complex V: ATP5PD) and MOM (PGAM5) [22] were identified to be interacted with TIPE3, indicating that TIPE3 were expressed in mitochondria (Fig. 3B). Metascape was performed to annotate the protein list and showed that the interactors were primarily enriched in mitochondrial functions, such as oxidative phosphorylation, apoptotic signaling pathway, response to oxidative stress (Fig. 3C). Respiratory chain complexes I interaction modes of the TIPE3 interactors was established by the PPI network (Supplementary Fig. 9).

As ETC complex drives oxidative phosphorylation (OXPHOS) to produce ATP and mitochondrial ROS that is the central of all cellular processes [23], then, one representative protein of each ETC complex subunits identified by co-IP/MS was validated using co-IP/WB. We confirmed that NDUFV2 (Complex I), MT-CO2 (Complex IV), and ATP5H (Complex V) were interacted with TIPE3 (Fig. 3D). Immunofluorescence staining illustrated that TIPE3 was co-localized with NDUFV2, MT-CO2 and ATP5H (Fig. 3E). To examine the subcellular organelle localization of TIPE3 in HNSCC, mitochondria isolation assay was applied. Western blotting assay confirmed that TIPE3 expressed in both the cytoplasm and mitochondria in TIPE3 overexpression cells (Fig. 3F). Comparing the pathways differences between TIPE3^high^ and TIPE3^low^ HNSCC patients using ssGSEA analysis, revealed that TIPE3^high^ specimens exhibited higher expressions of genes that involved in mitophagy, hypoxia, mitochondrial fission and apoptosis, and lower expressions of mitochondrial biogenesis genes (Fig. 3G). Genes correlated with TIPE3 was enriched in apoptosis activation (Fig. 3H). Therefore, these findings implied that TIPE3 might display its tumor suppression effects via interacting with ETC complex and inducing mitochondria stress in HNSCC cells.

### TIPE3 triggers mitochondria dysfunction in HNSCC cells

**Fig. 4:**
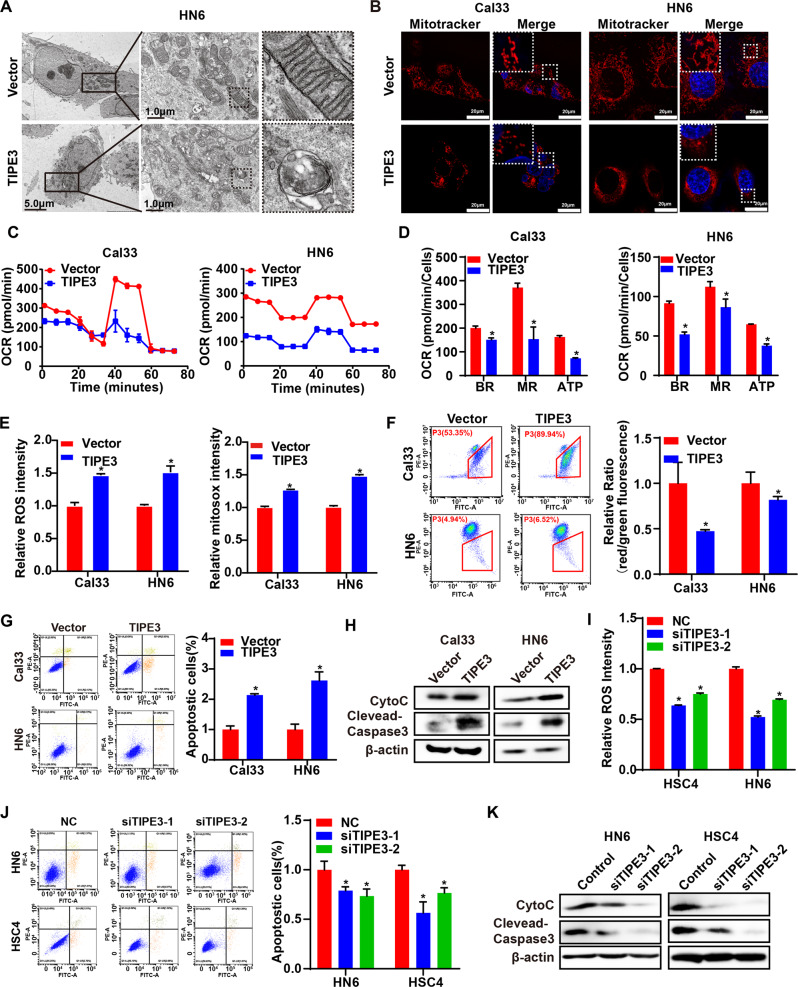
TIPE3 triggers mitochondrial damage and dysfunction. The mitochondrial stresses induced by TIPE3 were evaluated in Cal33 cells with vector or TIPE3 overexpression. **A** Representative TEM imaging of mitochondria. **B** Representative fluorescence imaging of mitochondria (red). **C**, **D** Oxygen consumption rate (OCR) of mitochondria ETC complex. **E** The cellular ROS and mtROS were determined with DCFH-DA and MitoSOX staining respectively. **F** The mitochondrial membrane potential was assessed by JC-1 staining. **G** Annexin-V-FITC/PI analysis of cell apoptosis. **H** WB analysis of cytoC, cleaved-caspase3, and β-actin expressions. **I**–**K** The si-NC or siTIPE3s transiently transferred into HN6 and HSC4 cells were used to examine the mitochondrial stresses suppressed by knocking down TIPE3 in HNSCC cells. **I** The cellular ROS was detected using DCFH-DA. **J** Annexin-V-FITC/PI analysis of cell apoptosis. **K** WB analysis of cytoC, cleaved-caspase3, and β-actin expressions. Mean ± s.d.; *P < 0.05; Student’s t-tests.

To further explore the roles of TIPE3 in mitochondrial stress, we compared the mitochondria morphology alterations between TIPE3 overexpression cells and vector cells using transmission electron microscope and Mitotracker Red staining assay. In comparation to the long mitochondria of vector cells, mitochondria became fragmented, round, and lost cristae in TIPE3 overexpression cells (Fig. 4A, B). Seahorse extracellular flux analyzer observed that the oxygen consumption rate (OCR) of basal and maximal respiration and ATP production were attenuated in TIPE3 overexpression cells, indicating the reduced OXPHOS and dysfunction of ETC complex [24] induced by TIPE3 (Fig. 4C, D). Mitochondrial and cytosolic ROS were accumulated (Fig. 4E), while the mitochondria membrane potential determined by JC-1 was decreased in TIPE3 overexpression cells (Fig. 4F), implying TIPE3 enhanced mitochondrial membrane depolarization and permeabilization [25]. Meanwhile, TIPE3 accelerated apoptosis and promote apoptotic molecules cytochrome c (cytoC) and cleaved-caspase3 expressions (Fig. 4G, H). Moreover, silencing TIPE3 attenuated ROS accumulation, and reduced apoptotic and its biomarkers expressions (Fig. 4I–K). Hence, those data demonstrated that TIPE3 triggered ETC and mitochondria dysfunction-induced apoptosis in HNSCC cell.

### TIPE3 induces mitochondria dysfunction through recruiting PGAM5-DRP1-BAX signaling in HNSCC cells

**Fig. 5:**
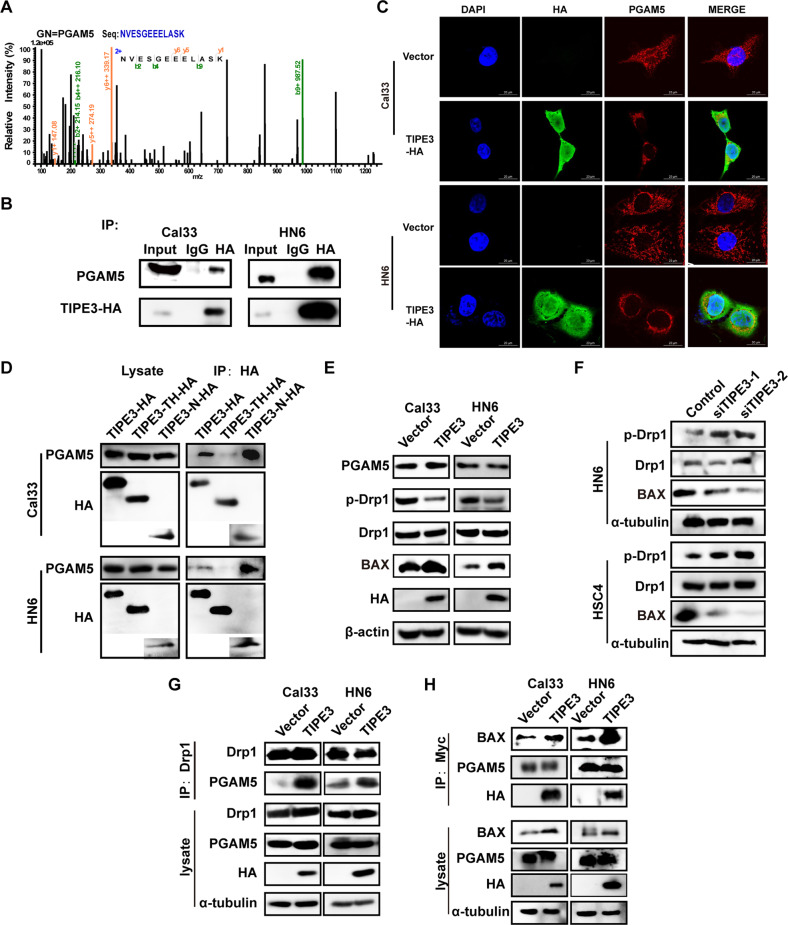
TIPE3 induces mitochondria stress via promoting PGAM5 dephosphorylating p-DRP1. **A** MS of PGAM5 proteins identified by Co-IP. **B** Physical interactions between TIPE3-HA and PGAM5 were examined via Co-IP/WB in Cal33 and HN6 cells with TIPE3-HA overexpression. **C** Immunofluorescence imaging of co-localization of TIPE3-HA and PGAM5. **D** Physical interactions between full-length TIPE3-HA, truncated TIPE3 (trTIPE3), NT region (NT), and PGAM5 were examined via Co-IP/WB in Cal33 and HN6 cells with TIPE3-HA overexpression. **E**, **F** Expressions of PGAM5, p-DRP1^ser637^, DRP1, BAX, HA, β-actin, and α-tubulin were determined by WB. **G**, **H** Physical interactions between DRP1 and PGAM5 (**G**), PGAM5 and BAX (**H**) were examined via Co-IP/WB in Cal33 and HN6 cells with vector or TIPE3-HA overexpression.

Next, we explored the mechanism of mitochondria dysfunction induced by restoring TIPE3. According to the interactors identified by co-IP/MS assay, PGAM5 (phosphoglycerate mutase family member 5, a mitochondrial phosphatase), which plays crucial roles in mitochondrial dynamics and apoptosis [26, 27], caught our attention (Fig. 5A). The co-IP/WB and immunofluorescence authenticated that TIPE3 could interact with and co-localized with PGAM5 in TIPE3 overexpression cells (Fig. 5B, C). TIPE3 contains two functional domains, one is the highly homologous TH domain involved in binding with lipids, the other is the unique NT region that is essential for regulating cell growth and survival [28]. To determine which domain is interacted with PGAM5, we constructed two truncated TIPE3 variant plasmids: one contains TH domain with NT region lacking, the other only has a truncated NT region. After deleting NT region, the interaction of trTIPE3 and PGAM5 became undetectable, while NT region alone showed a strong interaction with PGAM5 (Fig. 5D). Then, we examined the influence of TIPE3 on PGAM5 expression. We found that TIPE3 had no obvious effect on PGAM5 mRNA and protein levels (Fig. 5E, Supplementary Fig. 10).

The PGAM5-BAX-DRP1 complex play critical roles in apoptosis. Upon apoptosis induction, PGAM5 could activate BAX accumulating and inserting to the mitochondrial outer membrane. Meanwhile, PGAM5 dephosphorylated cytosolic p-DRP1^S637^ and activate its GTPase activity. The apoptotic executioner protein BAX and the dynamin-like protein DRP1 co-localize at mitochondria cristae to trigger mitochondrial permeabilization and fragmentation [27, 29]. Hence, we detected the effect of TIPE3 on p-DRP1^S637^ and BAX. We found that overexpressing TIPE3 decreased the phosphorylation levels of p-DRP1^S637^ and induced BAX expressions, while knocking down TIPE3 acted on a contrary way (Fig. 5E, F). Co-IP/WB indicated that TIPE3 could interact with BAX but not DRP1 (Supplementary Fig. 11). Silencing TIPE3 decreased the interactions of TIPE3 with PGAM5 and BAX (Supplementary Fig. 12). Moreover, overexpressing TIPE3 obviously promoted PGAM5 interacting with DRP1 and BAX (Fig. 5G, H). Collectively, these results demonstrated that TIPE3 recruit PGAM5 to promote BAX and DRP1 accumulating at the mitochondrial cristae, which mediated MOMP, cristae remodeling, mitochondrial fragmentation, and apoptosis.

### TIPE3 inhibits tumor progression via PGAM5 in HNSCC

**Fig. 6:**
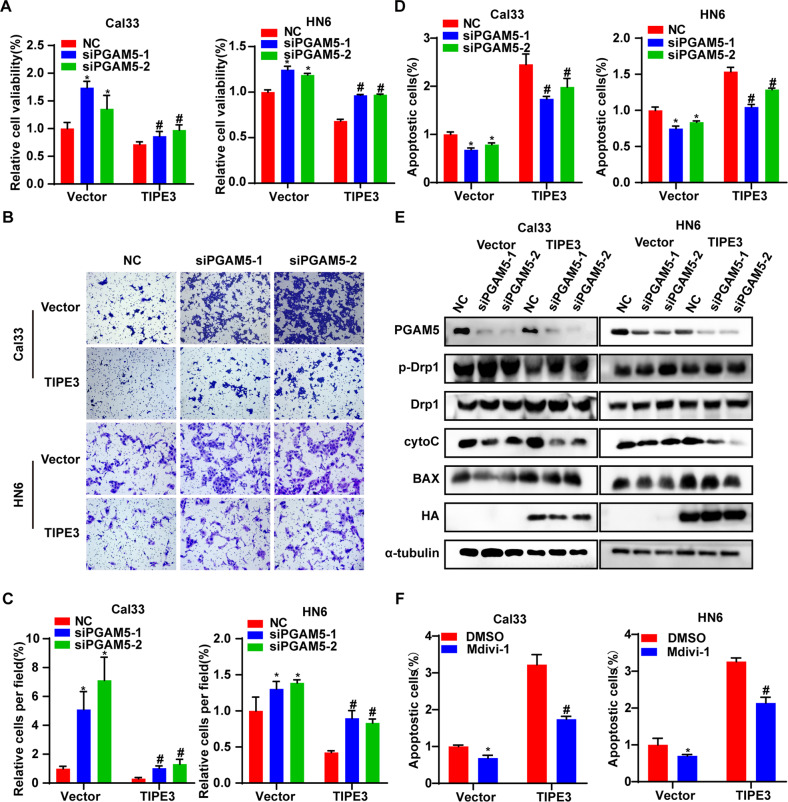
TIPE3 represses HNSCC progression via activating PGAM5-DRP1 signaling. **A**–**E** The si NC or si-PGAM5s was transfected in Cal33 and HN6 cells that stably overexpressed the vector or TIPE3. Cell viability was measured using CCK8 assay (**A**). Invaded cells were assessed using Transwell assay with Matrigel (**B**, **C**). Apoptotic cells were detected using Annexin-V-FITC/PI kit (**D**). The expression levels of PGAM5, p-DRP1^ser637^, DRP1, cytoC, BAX, HA, α-tubulin were determined using WB (**E**). **F** Cells with vector or TIPE3 stably overexpression were treated with Mdivi-1 (1 μm) or DMSO. The apoptotic cells were examined using Annexin-V-FITC/PI staining. Mean ± s.d.; *P < 0.05; Student’s t-tests.

To validate whether TIPE3 exerted the tumor suppression effect via PGAM5, we transiently knocked down PGAM5 expressions in TIPE3 or vector overexpression HNSCC cells (Supplementary Fig. 13). Silencing PGAM5 could remarkably rescue cell viability and invasion ability decreased by TIPE3 (Fig. 6A–C). Meanwhile, the elevated apoptotic cells and apoptosis biomarkers (cyto-C and BAX) and the decreased p-DRP1^S637^ in TIPE3 overexpression cells were reversed by siPGAM5s (Fig. 6D, E). Furthermore, treating TIPE3 and vector overexpression cells with Mdivi-1, a small-molecule inhibitor of Drp1 GTPases activity, obviously decreased the accumulation of apoptotic cells triggered by TIPE3 (Fig. 6F). Hence, those results demonstrated that TIPE3 induced mitochondria stress and apoptosis via PGAM5 in HNSCC cells.

### TIPE3 suppresses HNSCC tumor growth and metastasis in vivo

**Fig. 7:**
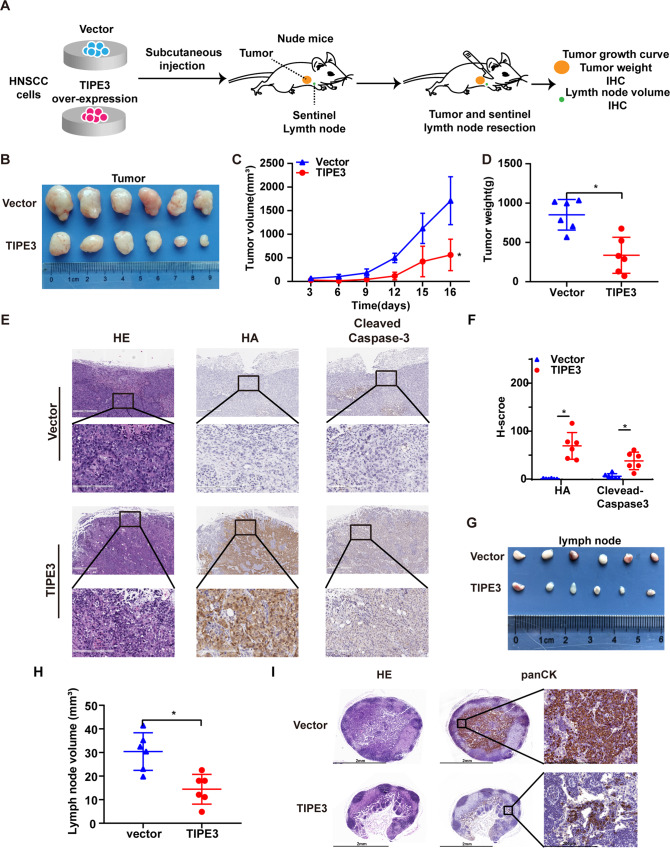
TIPE3 represses HNSCC tumor growth and metastasis in vivo. **A** HN6 cells stably overexpressed the vector or TIPE3 (*n* = 6 per group) were injected into the subcutaneous of mice to construct axillary lymph node metastasis models. **B** Representative images of primary tumors. **C**, **D** The volumes (**C**) and weights (**D**) of primary tumors. **E**, **F** Representative images (**E**) and quantification (**F**) of the expressions of HA, PGAM5, and cleaved-caspase3 in primary tumors determined by IHC staining. **G**, **H** Representative images (**G**) and quantification (**H**) of the average volumes of inguinal lymph nodes. **I** IHC staining of pan-cytokeratin-positive tumor cells in inguinal lymph nodes. Mean ± s.d.; *P < 0.05 compared with vector; Student’s t-tests.

Finally, the biological roles of TIPE3 in HNSCC cells in vivo were determined using a subcutaneous tumor growth and axillary lymph node metastasis nude mice model. After 6 weeks’ growth, the primary subcutaneous tumor and axillary lymph nodes were obtained (*n* = 6 per group, Fig. 7A). Compared with those in the vector group, primary tumors with TIPE3 overexpression group had slower growth rates, smaller volumes and lower weights (Fig. 7B–D). The IHC staining also revealed that cleaved-caspase-3 was highly expressed in TIPE3 overexpression primary tumors (Fig. 7E, F). The lymph nodes in the TIPE3 overexpression group showed smaller volumes and fewer pan-cytokeratin-positive tumor cells than those in the vector group (Fig. 7G–I). Taken together, these findings demonstrated that TIPE3 repressed HNSCC cells growth and lymph node metastasis in vivo.

## Discussion

**Fig. 8:**
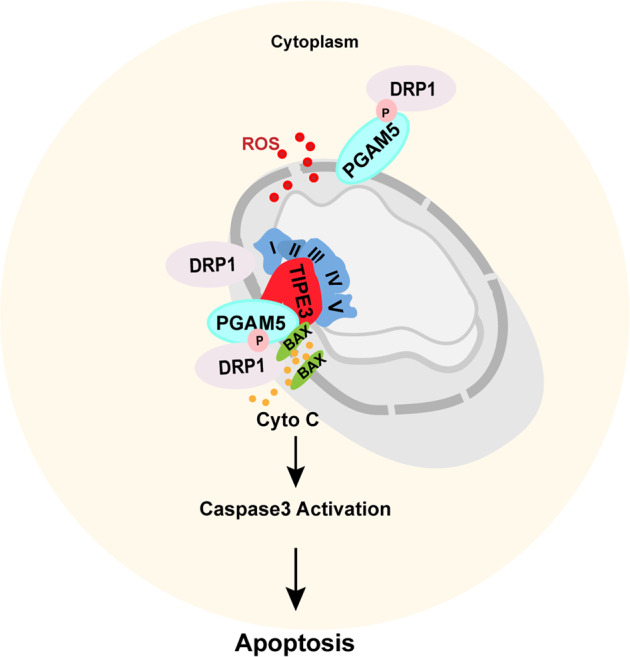
Schematic model of the role of TIPE3 in regulating mitochondrial stress in HNSCC cells. HNSCC cells downregulate TIPE3 via promoter CpG island hypermethylation to avoid detrimental mitochondrial stress during rapid proliferation. Overexpressing of TIPE3 increases PGAM5 targeting mitochondrial membrane to recruit BAX and facilitate p-DRP1^ser637^ dephosphorylation and activation at ETC complex site of cristae, leading to mitochondrial membrane fragmentation, ROS accumulation, and cytochrome-c releasing, which ultimately induced HNSCC cell apoptosis.

Mitochondria are essential organelles in balancing oxidative stress and cell death during tumor cell proliferation. Understanding the mechanisms of tumor cells defending mitochondrial stress to avoid apoptosis will be critical for the next generation of cancer therapeutics. Here, downregulation of TIPE3 due to its promoter hypermethylation was identified to be critical in balancing tumor growth and apoptosis during HNSCC progression. Low TIPE3 levels correlated with locally advanced tumors and poor survival of HNSCC patients. TIPE3 induced mitochondria dysfunction, decreased cell viability, and promoted apoptosis in vitro and in vivo. Mechanistically, TIPE3 expressed in mitochondria interacted with MIM subunits ETC complex and MOM protein PGAM5, promoted PGAM5 recruiting BAX and DRP1 to the MOM followed by forming pores, constricting and splitting the mitochondria, which ultimately triggered ETC complex dysfunction, ROS accumulation, mitochondrial fragmentation, and apoptosis (Fig. 8).

TIPE3, as the newest member of TIPEs, only few reports are available on its biological functions. Svetlana A. Fayngerts and his colleagues firstly reported that, TIPE3 transferred lipid second messengers through its TH domain and shuttling them through the aqueous phase, which might enhance the activity of phosphoinositide-modifying enzymes such as PI3K [28]. Yet, TIPE3 promoted cell progression was not relied on the TH domain but its unique NT region, truncating which induced tumor suppression effect in lung carcinoma cells, bladder carcinoma cells, and colorectal adenocarcinoma cells [28]. Based on these characteristics, full-length TIPE3 had been confirmed to promote several cancer progression, including cervical cancer [28], breast cancer [30], and stomach cancer [31]. However, we previously identified that TIPE3 was hypermethylated and downregulated in several cancers, including bladder cancer, colon and rectal cancer, lung cancer, prostate cancer, and HNSC in TCGA. For the first time, we found that overexpressing full-length TIPE3 acted as a tumor suppressor and suppressed nasopharyngeal carcinoma (NPC) cells progression in vitro and in vivo [17]. However, the mechanism of its tumor suppression effect is unknown. In the present study, we comprehensively analyzed the expression alterations of TIPEs and their correlations with HNSCC clinical outcomes using public multicenter cohorts with large samples. We confirmed the downregulation of TIPE3 via promoter hypermethylation in tumor tissues might be a critical event during HNSCC tumorigenesis. Overexpressing full-length TIPE3 decreased HNSCC cell viability and induced apoptosis.

Mitochondria play a central role in supporting tumor growth via providing energy and oncometabolites, modulating calcium homeostasis, and balancing ROS generation and scavenging. Paradoxically, mitochondria also display a central role in apoptotic cell death. Upon mitochondrial apoptosis induction, membranes permeabilization and its downstream cytochrome c release and subsequent caspase activation usually commits a cell to die [32]. In the present study, we showed that restoring TIPE3 interacted with the MIM ETC complex, disrupted the mitochondria membrane and enhanced permeability (represented as obscure cristae, mitochondrial membrane potential depolarization, and increased round mitochondria [25, 33]), and induced mitochondria dysfunction (represented as OXPHOS and ATP generation decrease and ROS accumulation [25, 33]) in HNSCC cells. Furthermore, the apoptotic proteins (cyto-c, BAX, and cleaced-caspase-3) and apoptotic cells were elevated by TIPE3. Hence, these evidences authorize that TIPE3 represses HNSCC progression through inducing mitochondrial apoptosis.

A mitochondrial phosphatase PGAM5 was identified to be an interactor of TIPE3 by our co-IP/MS assay. Although the ultra-micro-positioning of PGAM5 variants and their roles in regulating cell death remains unclear, PGAM5 has been reported to assist apoptotic proteins to initiate apoptosis via several ways, including acitivating BAX and recruiting DRP1 to induce mitochondrial fission and mitochondrial outer membrane permeabilization evoking [18, 34–36]. Our data illustrated that TIPE3 interacted with PGAM5 via its NT region and promoted PGAM5 dephosphorylating the cytosolic p-DRP1^S637^ and accumulating BAX. Inhibiting either PGAM5 or DRP1 activity rescued the tumor suppression effect induced by TIPE3. Considering TIPE3 was reported to bind to the phosphoinositides of cytomembrane lipid bilayer via its TH domain, thus we proposed that TIPE3 acted as a bridge of MIM ETC complex and MOM protein PGAM5. We hypothesized that, TIPE3 used its TH domain banding to phospholipid substrates and ETC complex of the cristae and its NT region banding to PGAM5 to recruit and activate apoptotic inducers, which need to be further investigated by structural biology study (Fig. 8).

In summary, we demonstrate that the downregulation of TIPE3 via its promoter hypermethylation is the main molecular event of TIPE family during HNSCC tumorigenesis. HNSCC cells repress TIPE3-PGAM5-DRP1-BAX triggering mitochondrial stress and apoptosis to promote tumor growth and metastasis. We establish that TIPE3 is an attractive therapeutic target for HNSCC patients.

## Supplementary information

reproducibility checklist

Supplemental material

Unedited gel for figures

## Data Availability

The data of this research are available from the corresponding authors on reasonable request.

## Materials availability

The materials of this research are available from the corresponding authors on reasonable request.
