# Supplementary material for: TIPE3 represses head and neck squamous cell carcinoma progression via triggering PGAM5 mediated mitochondria dysfunction
Source: Cell Death Dis. 2023 Apr 6;14(4):251. doi: 10.1038/s41419-023-05775-3 (PMC10079926; doi:10.1038/s41419-023-05775-3)
Supplement: Supplementary file 2 — Supplemental material [file 41419_2023_5775_MOESM2_ESM.docx]

**TIPE3 represses head and neck squamous cell carcinoma progression via triggering PGAM5 mediated mitochondria dysfunction**

Wei Chen,^1,2,3*^ Xijuan Chen,^1,2,3*^ Lixuan Wang,^1,2,3*^ Rongchun Yang,^1,2,3^ Weilin Zhang,^1,2,3^ Siyuan Zhang,^1,2,3^ Juan Xia,^1,2,3^ Bin Cheng,^1,2,3†^ Tong Wu,^1,2,3†^ Xianyue Ren^1,2,3†^


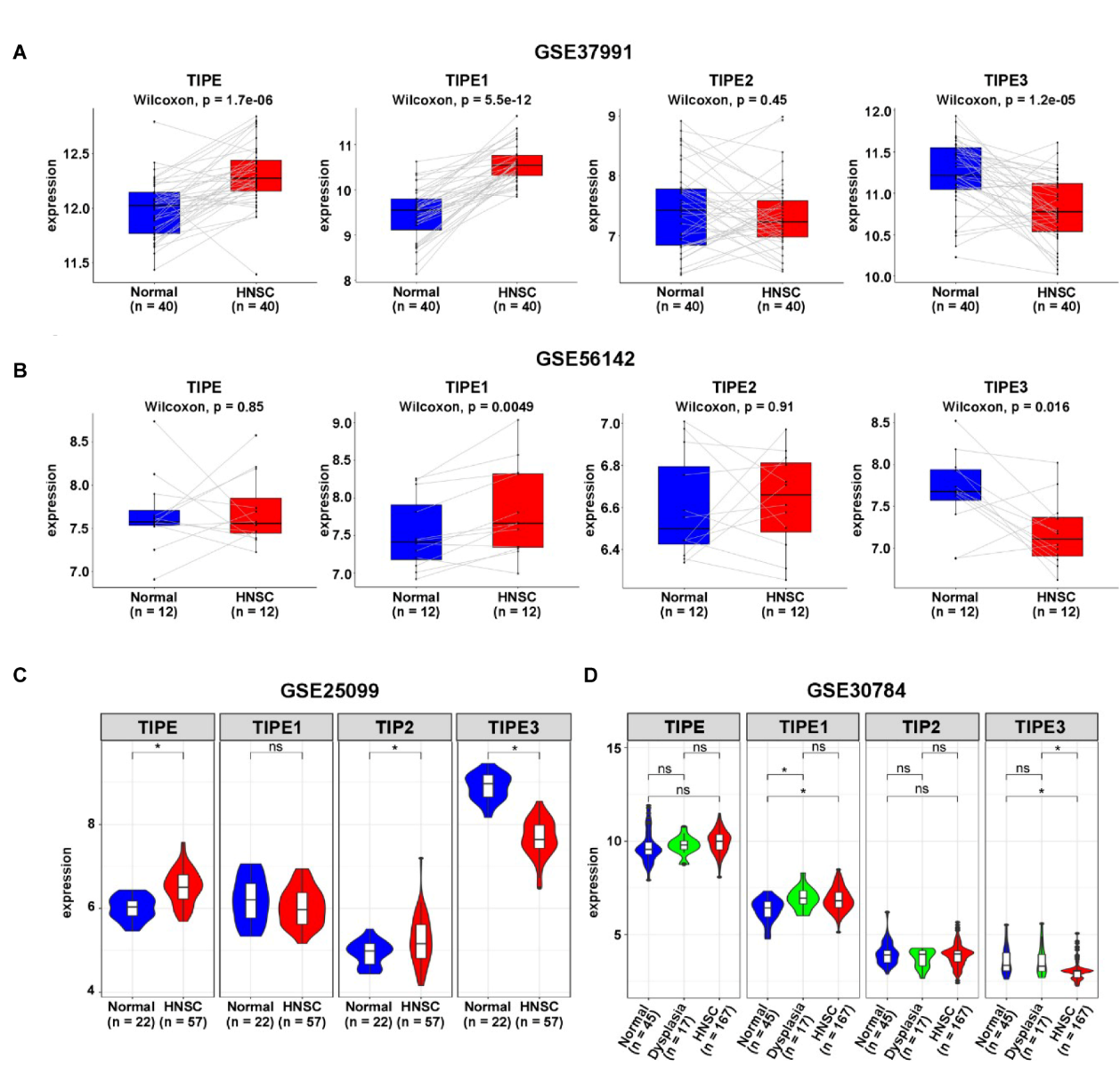


**Supplementary Figure 1. TIPE3 is downregulated in HNSCC.** (A-D) The mRNA levels of TIPEs in normal epithelia tissues and in HNSCC tissues in GSE37991 (A), GSE56142 (B), GSE25099 (C) and GSE30784 (D) datasets.


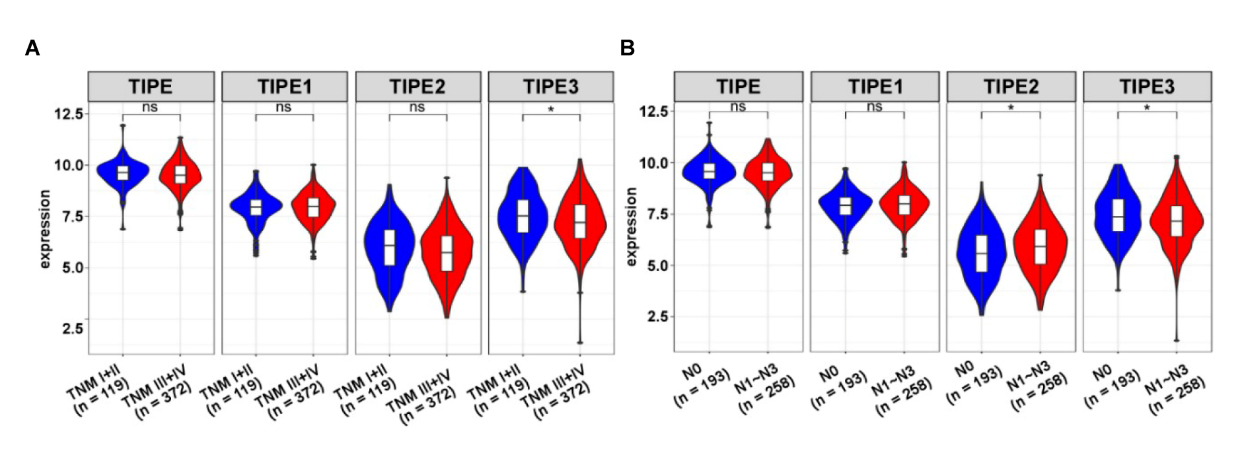


**Supplementary Figure 2. TIPE3 is associated with the aggressiveness of HNSCC tumors.** (A-B) the mRNA levels of TIPEs in patients with early-stage (TNM I-II) and late-stage (TNM III-IV) (A), and those with or without lymph node metastasis (B) based on the TCGA-HNSC cohort.


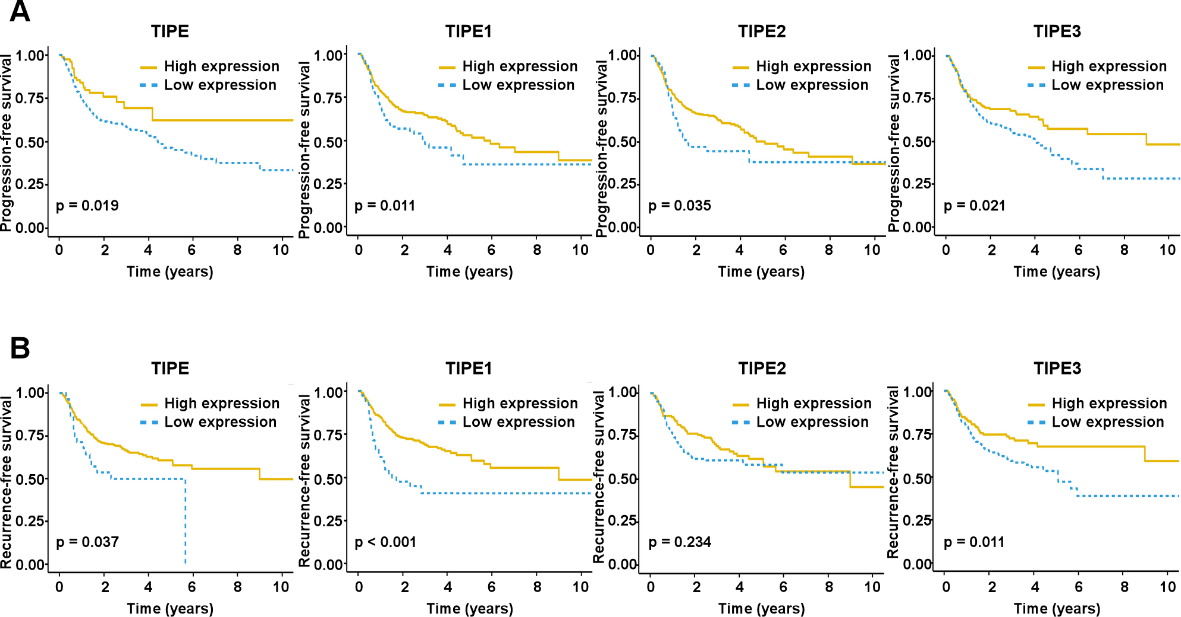


**Supplementary Figure 3. TIPE3 downregulation is associated with poor clinical outcomes of HNSCC patients.** (A) progression-free survival (PFS) and (B) recurrence-free survival (RFS) in HNSCC patients in the TCGA-HNSC cohort.


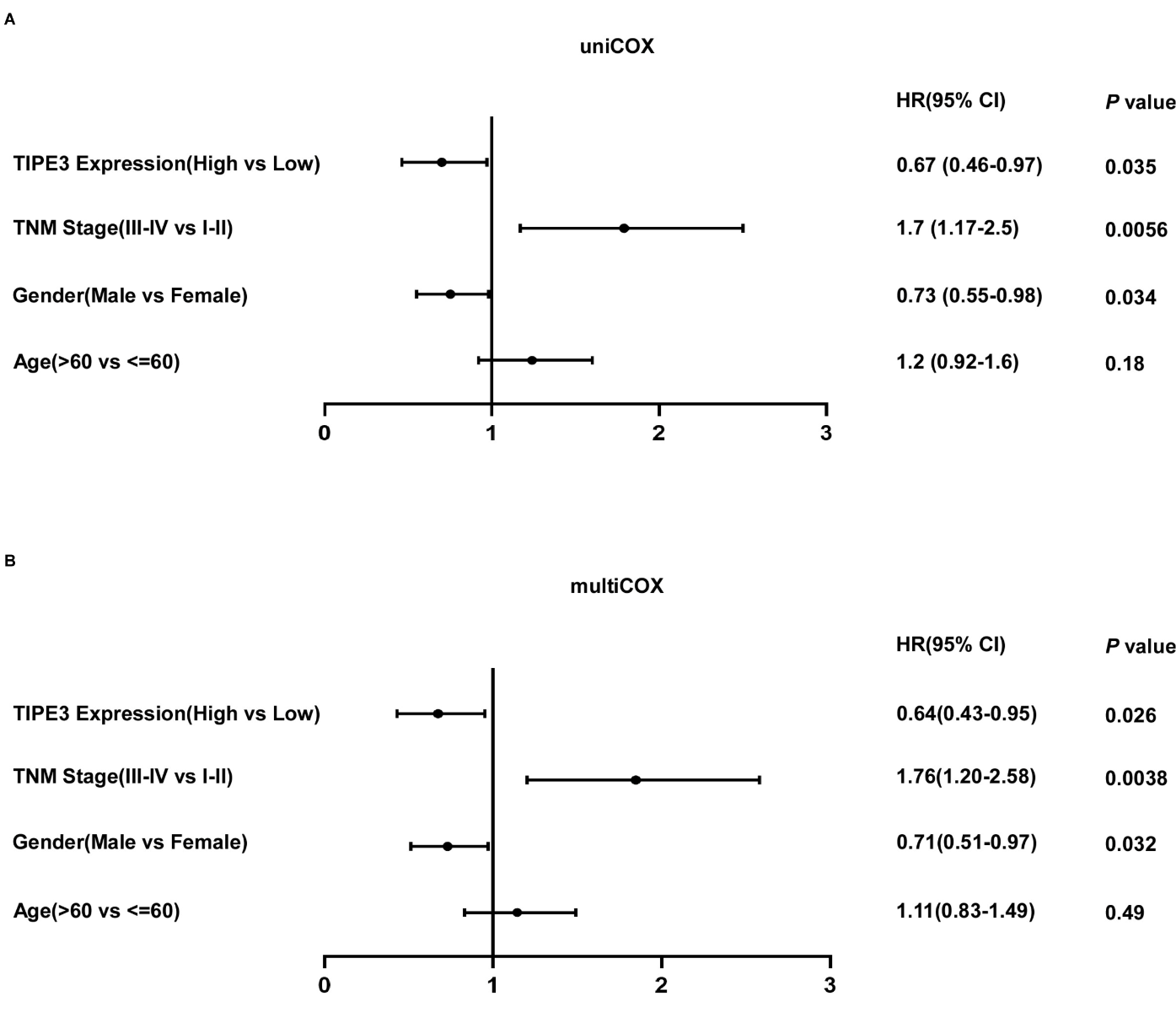


**Supplementary Figure 4. TIPE3 downregulation is associated with poor clinical outcomes of HNSCC patients.** Univariate (A) and Multivariate (B) COX analysis of the TIPE3 and clinicopathological characteristics with overall survival based on the TCGA-HNSC cohort.


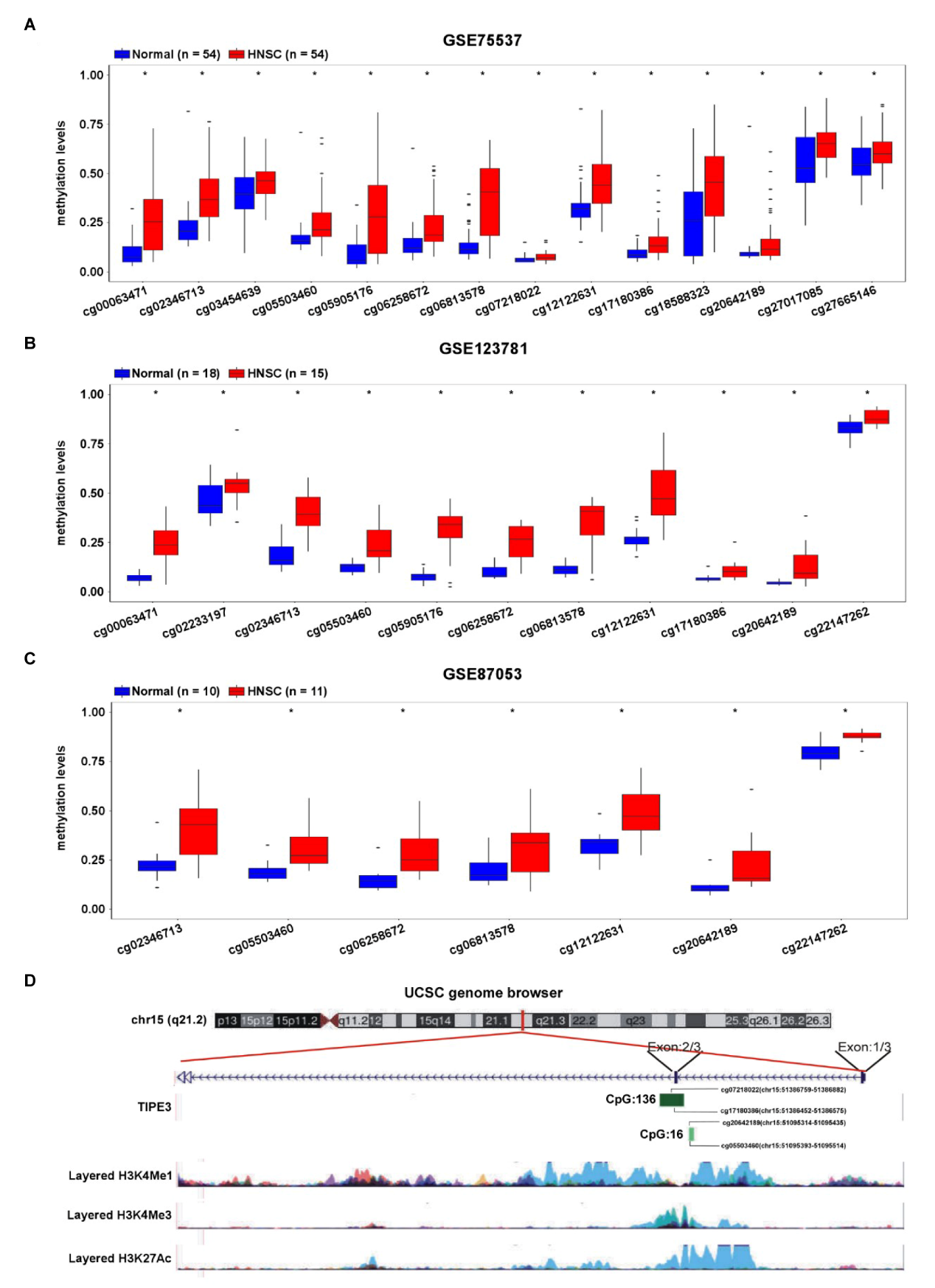


**Supplementary Figure 5. TIPE3 is hypermethylated in HNSCC.** (A-C) Methylation levels of TIPE3 in normal and HNSCC tissues in GSE75537 (A), GSE123781 (B) and GSE87053 (C). (D) The genome features of TIPE3 observed using the UCSC genome browser.


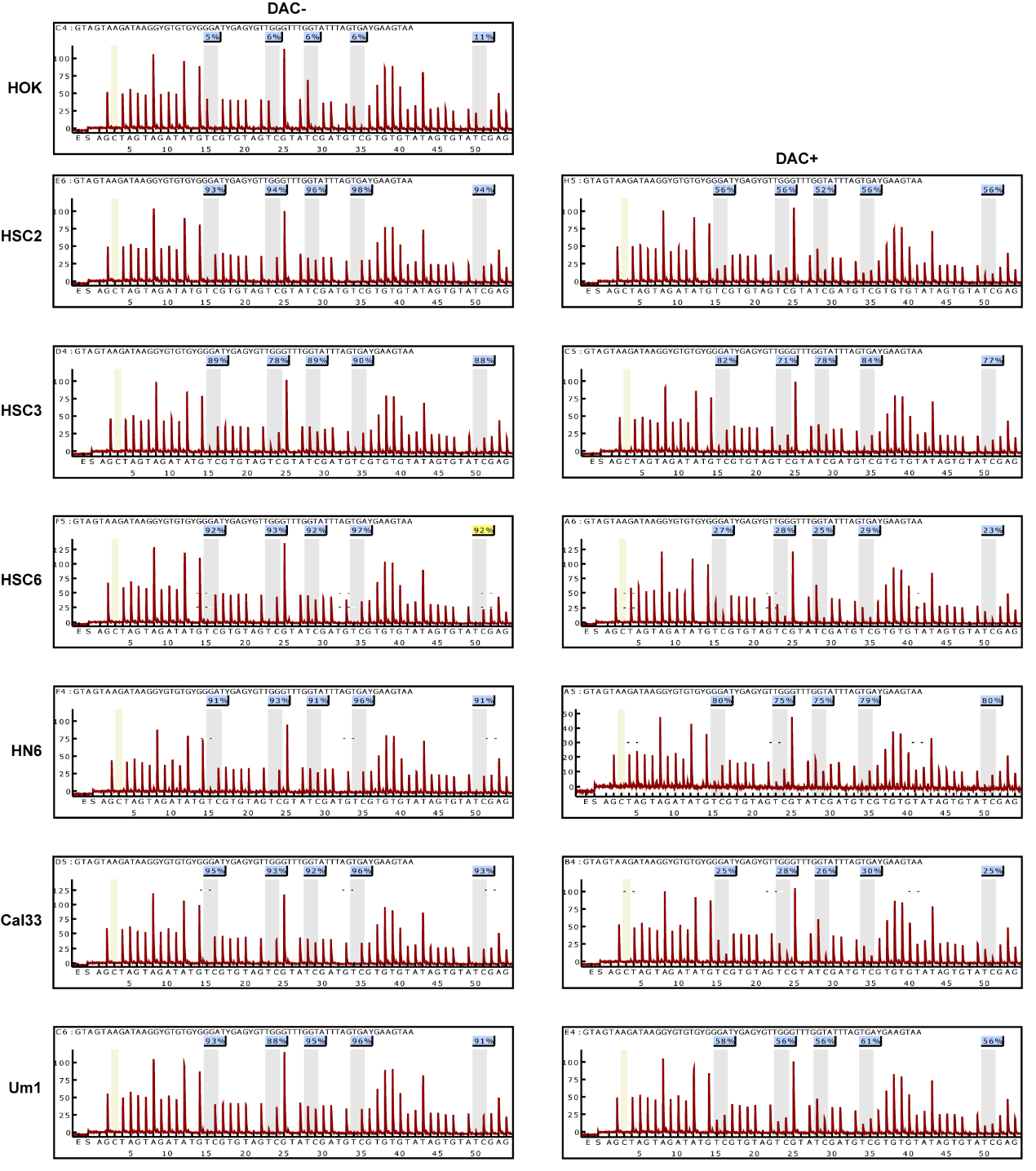


**Supplementary Figure 6. TIPE3 is hypermethylated in HNSCC.** Bisulfite pyrosequencing analysis of the HOPX promoter region in HOK and HNSCC (HSC2, HSC3, HSC6, HN6, Cal33 and UM1) cell lines.


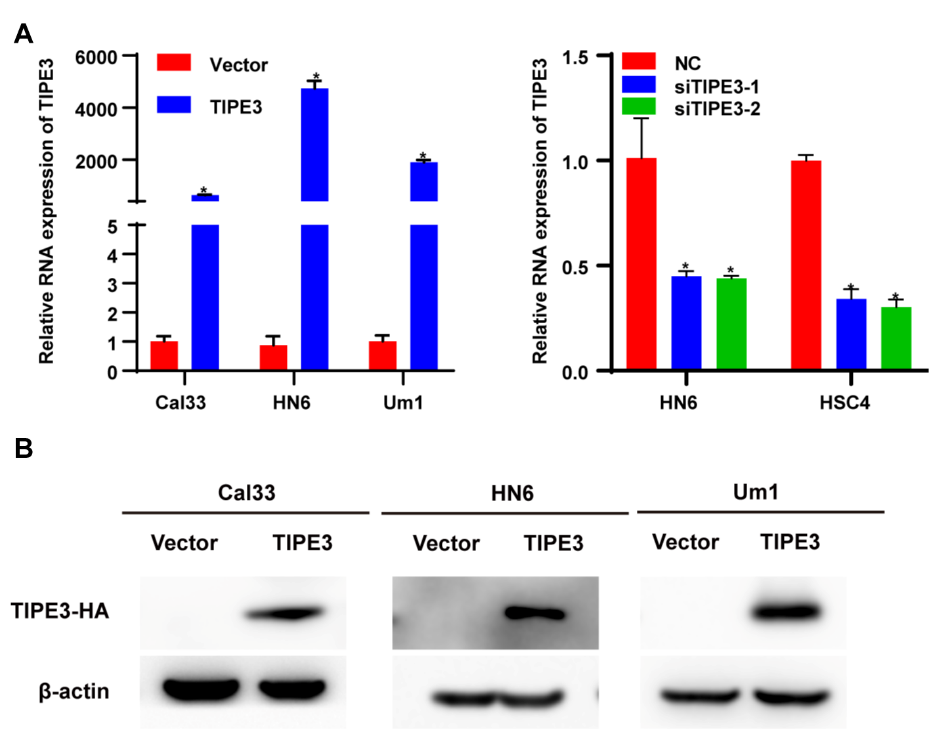


**Supplementary Figure 7. Construction of HNSCC cells with TIPE3 stably overexpression or with TIPE3 transiently silencing.** The qPCR(A) and Western blot (B) were performed to examine the transfection efficiencies of TIPE3 overexpression or knockdown in HNSCC cell lines.


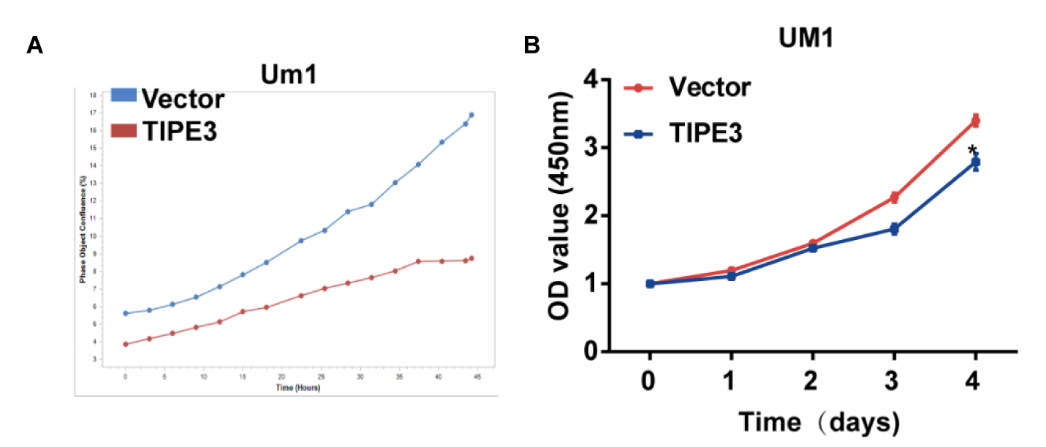


**Supplementary Figure 8. TIPE3 overexpression inhibited the proliferation of UM1 cells.** Live-cell analysis system (A) and CCK8 (B) were used to detect the effect of TIPE3 overexpression on proliferation in UM1.


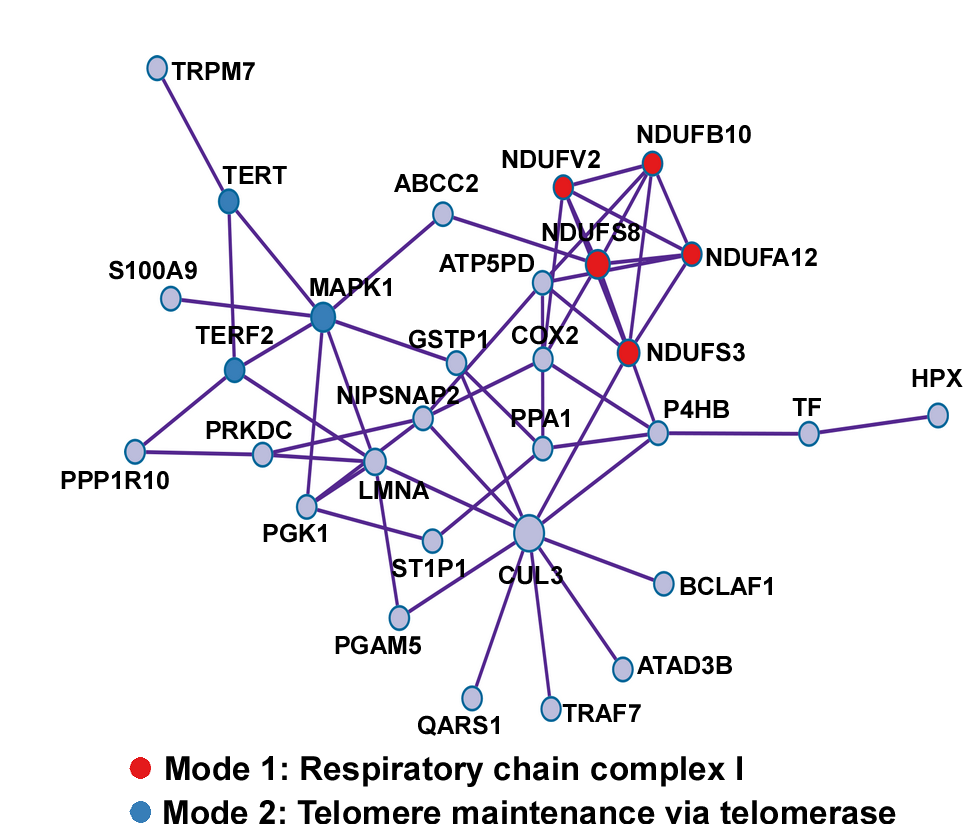


**Supplementary Figure 9. Respiratory chain complexes I interaction modes was established by the PPI interaction network in Metascape.**


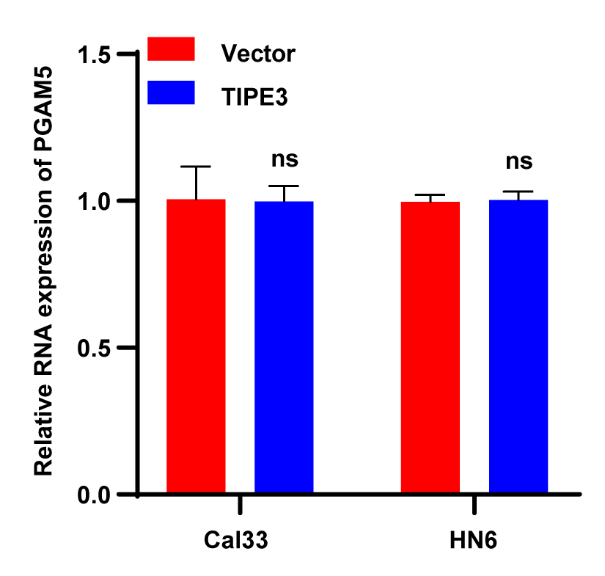


**Supplementary Figure 10. The qPCR analysis showed that TIPE3 had no obvious effect on PGAM5 mRNA levels.**


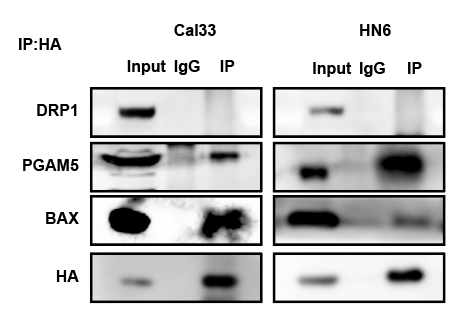


**Supplementary Figure 11. The interactions of TIPE3-HA and DRP1 or BAX were determined using Co-IP/WB assay.**

**
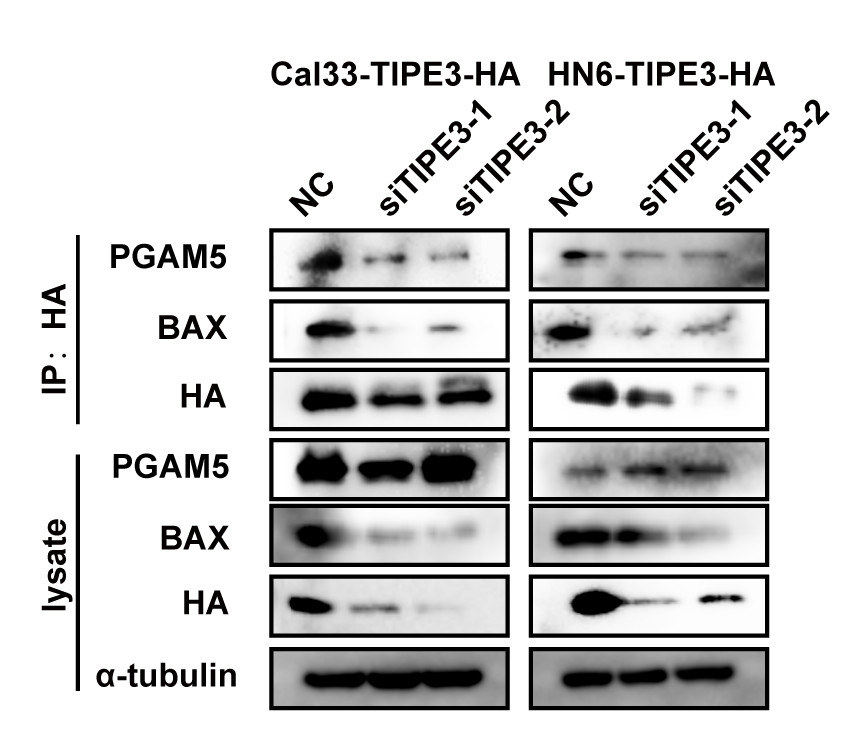
**

**Supplementary Figure 12. After knocking down the expression levels of TIPE3 in Cal33 and HN6 cells with TIPE3-HA stably overexpressed, Co-IP/WB was performed. The interactions between TIPE3-HA, PGAM5 and BAX were examined.**


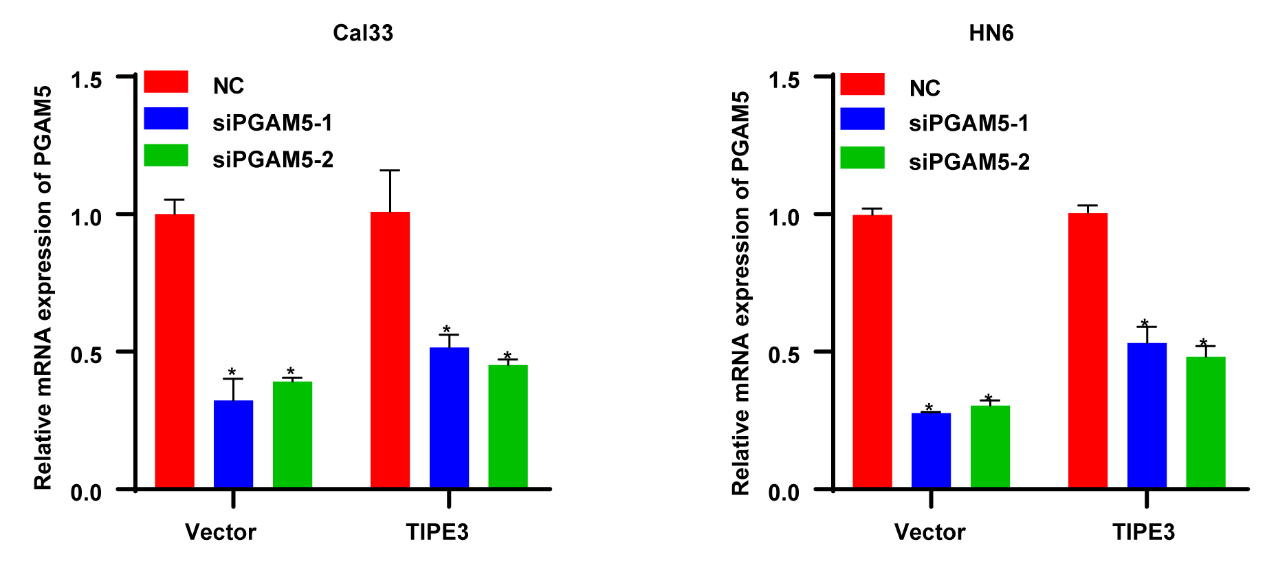


**Supplementary Figure 13. The efficiencies of silencing PGAM5 in HNSCC cell lines were detected using qPCR.**

**Supplementary Table S1. Primers used in this study.**

| Gene | Sequence (5' to 3') |
| --- | --- |
| **BSP PCR primers** | |
| TIPE3-F1 | GGGGAGATGATAGTTGGAT |
| TIPE3-R1-Bio | CTTACCCCACCTACCCTAC（5’-Biotin） |
|  |  |
| **BSP sequencing primer** | |
| TIPE3-S1 | TGGATTGTTGTGGAAA |
|  |  |
| **Real-time RT-PCR primers** | |
| TIPE3-F | ATGGATTCGGATTCCGGGG |
| TIPE3-R | TTAAAGGACTTTCTCATCTAGCAAC |
| PGAM5-F  PGAM5-R  GAPDH-F  GAPDH-R | CTCTCCCTCAATAATGGCAG  CAGGATCGAGTGATCTTGTC  GCACCGTCAAGGCTGAGAAC TGGTGAAGACGCCAGTGGA |
| **siRNA sequences** |  |
| siRNA-NC | 5’-CCATGACCATTGTCAGCTT-3’ |
| siTIPE3-1  siTIPE3-2 | 5’-GGAACGTGCTCTCCAATCT-3’  5’-CGCAGCATGGATTCGGATT-3’ |

**Supplementary Table S2. Antibodies used in this study.**

| Antibodies | Company | Catalog no. | Dilution |
| --- | --- | --- | --- |
| **Western blotting** | | | |
| PGAM5 | Proteintech | 28445-1-AP | 1:1000 |
| DRP1 | Proteintech | 12957-1-AP | 1:1000 |
| TOM20 | Proteintech | 11802-1-AP | 1:2000 |
| BAX | proteintech | 50599-2-Ig | 1:1000 |
| Phospho-DRP1(Ser637) | CST | 4867S | 1:1000 |
| HA  Caspase3  Clevead-caspase3  CytochromeC  ATP5H  NDUFV2  MTCO2 | CST  CST  CST  Proteintech  Proteintech  Proteintech  Proteintech | 3724S  9665S  9664S  66264-1-Ig  17589-1-AP  15301-1-AP  55070-1-AP | 1:1000  1:1000  1:500  1:1000  1:1000  1:1000  1:1000 |
| β-actin  GAPDH  α-tubulin | Santa Cruz  Proteintech  Proteintech | Sc-47778  60004-1-Ig  11224-1-AP | 1:1000  1:1000  1:1000 |
| Mouse | CST | 7076 | 1:5000 |
| Rabbit | CST | 7074 | 1:5000 |
| **Immunohistochemistry** |  |  |  |
| PGAM5 | Proteintech | 28445-1-AP | 1:100 |
| HA  Clevead-caspase3 | CST  CST | 3724S  9664S | 1:200  1:100 |
| **Immunofluorescence** |  |  |  |
| PGAM5  PGAM5  NDUFV2  HA | Proteintech  Abcam  Proteintech  CST | 28445-1-AP  ab126534  15301-1-AP  3724S | 1:50  1:50  1:50  1:100 |
| ATP5H | Proteintech | 17589-1-AP | 1:100 |
| MTCO2 | Proteintech | 55070-1-AP | 1:100 |
| goat Anti-rabbit, Alexa Fluor® 594 IgG secondary antibody | ThermoFisher | R37117 | 1:1000 |
| goat Anti-mouse, Alexa Fluor® 488 IgG secondary antibody | ThermoFisher | F2761 | 1:1000 |
| **Co-Immunoprecipitation** |  |  |  |
| HA | CST | 3724S | 3μg |
| MYC |  |  | 3μg |
| DRP1 | Proteintech | 12957-1-AP | 3μg |
| IgG | proteintech | B900610 | 3μg |

**Supplementary Table 3. The list of proteins that interacted with TIPE3-HA identified by co-IP/MS.**

| **prot_desc** | **prot_score** | **prot_matches** |
| --- | --- | --- |
| Immunoglobulin heavy variable 1-45 GN=IGHV1-45 | 292 | 7 |
| RNA-binding protein EWS GN=EWSR1 | 119 | 4 |
| Immunoglobulin kappa variable 2-28 GN=IGKV2-28 | 115 | 2 |
| RNA-binding protein FUS GN=FUS | 112 | 5 |
| Ras-related protein Rab-7a GN=RAB7A | 92 | 2 |
| Ras-related protein Rab-1B GN=RAB1B | 80 | 3 |
| Cleavage and polyadenylation specificity factor subunit 5 GN=NUDT21 | 75 | 2 |
| Lamina-associated polypeptide 2, isoforms beta/gamma GN=TMPO | 71 | 1 |
| 60S ribosomal protein L24 GN=RPL24 | 67 | 1 |
| U1 small nuclear ribonucleoprotein C GN=SNRPC | 65 | 1 |
| Immunoglobulin kappa variable 4-1 GN=IGKV4-1 | 65 | 2 |
| 60S ribosomal protein L15 GN=RPL15 | 63 | 1 |
| Tubulin beta-8 chain GN=TUBB8 | 63 | 2 |
| Plasminogen activator inhibitor 1 RNA-binding protein GN=SERBP1 | 53 | 2 |
| Serine/arginine-rich splicing factor 7 GN=SRSF7 | 46 | 1 |
| Transmembrane emp24 domain-containing protein 9 GN=TMED9 | 46 | 1 |
| Protein FAM98A GN=FAM98A | 43 | 1 |
| Bleomycin hydrolase GN=BLMH | 41 | 1 |
| Skin-specific protein 32 GN=XP32 | 40 | 1 |
| Cytochrome c oxidase subunit 2 GN=MT-CO2 | 40 | 1 |
| Transmembrane protein adipocyte-associated 1 GN=TPRA1 | 38 | 3 |
| 60S ribosomal protein L29 GN=RPL29 | 37 | 1 |
| NADH dehydrogenase [ubiquinone] iron-sulfur protein 8, mitochondrial GN=NDUFS8 | 37 | 1 |
| ATP synthase subunit d, mitochondrial GN=ATP5PD | 36 | 1 |
| Immunoglobulin heavy constant gamma 2 GN=IGHG2 | 36 | 1 |
| Probable non-functional immunoglobulin heavy variable 3-16 GN=IGHV3-16 | 34 | 1 |
| NADH dehydrogenase [ubiquinone] flavoprotein 2, mitochondrial GN=NDUFV2 | 33 | 1 |
| 60S ribosomal protein L17 GN=RPL17 | 33 | 1 |
| Keratin, type I cytoskeletal 39 GN=KRT39 | 32 | 1 |
| Leucine-rich repeat-containing protein 10B GN=LRRC10B | 31 | 1 |
| Glutamine--tRNA ligase GN=QARS1 | 31 | 1 |
| Probable non-functional immunoglobulin kappa variable 2D-24 GN=IGKV2D-24 | 31 | 2 |
| Hypoxanthine-guanine phosphoribosyltransferase GN=HPRT1 | 31 | 1 |
| Telomerase reverse transcriptase GN=TERT | 29 | 1 |
| RNA-binding protein 8A GN=RBM8A | 29 | 2 |
| Deformed epidermal autoregulatory factor 1 homolog GN=DEAF1 | 28 | 1 |
| Histone H2A.Z GN=H2AZ1 | 28 | 1 |
| C-C motif chemokine 16 GN=CCL16 | 28 | 1 |
| Growth hormone secretagogue receptor type 1 GN=GHSR | 28 | 1 |
| WD40 repeat-containing protein SMU1 GN=SMU1 | 27 | 1 |
| Glutathione S-transferase P GN=GSTP1 | 27 | 1 |
| Maestro heat-like repeat-containing protein family member 2B GN=MROH2B | 27 | 1 |
| Kinesin-like protein KIF20B GN=KIF20B | 25 | 1 |
| Dedicator of cytokinesis protein 5 GN=DOCK5 | 25 | 1 |
| ER membrane protein complex subunit 2 GN=EMC2 | 25 | 1 |
| Protein LSM14 homolog A GN=LSM14A | 25 | 1 |
| Immunoglobulin heavy variable 3-15 GN=IGHV3-15 | 25 | 1 |
| Coiled-coil domain-containing protein 87 GN=CCDC87 | 25 | 1 |
| Protein disulfide-isomerase GN=P4HB | 24 | 1 |
| NADH dehydrogenase [ubiquinone] 1 beta subcomplex subunit 10 GN=NDUFB10 | 24 | 1 |
| SWI/SNF-related matrix-associated actin-dependent regulator of chromatin subfamily D member 3 GN=SMARCD3 | 24 | 1 |
| Nuclear receptor subfamily 0 group B member 1 GN=NR0B1 | 24 | 1 |
| E3 ubiquitin-protein ligase TRAF7 GN=TRAF7 | 24 | 1 |
| Myozenin-2 GN=MYOZ2 | 23 | 1 |
| ADP-ribosylation factor GTPase-activating protein 1 GN=ARFGAP1 | 23 | 1 |
| Selenocysteine insertion sequence-binding protein 2-like GN=SECISBP2L | 23 | 1 |
| Protein bassoon GN=BSN | 22 | 1 |
| MOB kinase activator 1B GN=MOB1B | 22 | 1 |
| Peroxisomal acyl-coenzyme A oxidase 3 GN=ACOX3 | 20 | 1 |
| Dual specificity protein kinase CLK2 GN=CLK2 | 19 | 1 |
| NADH dehydrogenase [ubiquinone] 1 alpha subcomplex subunit 12 GN=NDUFA12 | 19 | 1 |
| Prostaglandin F2 receptor negative regulator GN=PTGFRN | 18 | 1 |
| Methylcytosine dioxygenase TET1 GN=TET1 | 18 | 1 |
| Transient receptor potential cation channel subfamily M member 7 GN=TRPM7 | 17 | 1 |
| Proheparin-binding EGF-like growth factor GN=HBEGF | 16 | 1 |
| Canalicular multispecific organic anion transporter 1 GN=ABCC2 | 16 | 1 |
| CDK5 regulatory subunit-associated protein 3 GN=CDK5RAP3 | 16 | 1 |
| Torsin-2A GN=TOR2A | 15 | 1 |
| Agrin GN=AGRN | 15 | 1 |
| DNA-directed RNA polymerase I subunit RPA2 GN=POLR1B | 15 | 1 |
| Scaffold attachment factor B1 GN=SAFB | 13 | 1 |
| Serine/arginine repetitive matrix protein 2 GN=SRRM2 | 384 | 10 |
| Non-POU domain-containing octamer-binding protein GN=NONO | 285 | 15 |
| Cullin-3 GN=CUL3 | 263 | 11 |
| RNA-binding protein 14 GN=RBM14 | 218 | 8 |
| Keratin, type II cytoskeletal 4 GN=KRT4 | 198 | 7 |
| KH domain-containing, RNA-binding, signal transduction-associated protein 1 GN=KHDRBS1 | 194 | 9 |
| Heterogeneous nuclear ribonucleoprotein M GN=HNRNPM | 187 | 4 |
| Treacle protein GN=TCOF1 | 185 | 5 |
| Tubulin alpha-1C chain GN=TUBA1C | 175 | 7 |
| Keratin, type I cytoskeletal 28 GN=KRT28 | 146 | 8 |
| Probable ATP-dependent RNA helicase DDX5 GN=DDX5 | 131 | 5 |
| ATP-dependent RNA helicase DDX1 GN=DDX1 | 124 | 5 |
| Nucleolar and coiled-body phosphoprotein 1 GN=NOLC1 | 105 | 9 |
| Serine/threonine-protein phosphatase PGAM5, mitochondrial GN=PGAM5 | 98 | 6 |
| U1 small nuclear ribonucleoprotein 70 kDa GN=SNRNP70 | 98 | 5 |
| Chloride channel CLIC-like protein 1 GN=CLCC1 | 95 | 2 |
| Keratin, type II cytoskeletal 71 GN=KRT71 | 92 | 5 |
| FRAS1-related extracellular matrix protein 2 GN=FREM2 | 92 | 4 |
| Heterogeneous nuclear ribonucleoprotein R GN=HNRNPR | 83 | 2 |
| Small nuclear ribonucleoprotein-associated proteins B and B~ GN=SNRPB | 77 | 5 |
| Polyadenylate-binding protein 4 GN=PABPC4 | 74 | 3 |
| Heat shock 70 kDa protein 4 GN=HSPA4 | 73 | 3 |
| Heterogeneous nuclear ribonucleoprotein Q GN=SYNCRIP | 73 | 3 |
| Cleavage and polyadenylation specificity factor subunit 6 GN=CPSF6 | 73 | 2 |
| Very-long-chain enoyl-CoA reductase GN=TECR | 66 | 2 |
| THO complex subunit 4 GN=ALYREF | 65 | 1 |
| Luc7-like protein 3 GN=LUC7L3 | 64 | 1 |
| Tubulin beta chain GN=TUBB | 62 | 4 |
| ATPase family AAA domain-containing protein 3B GN=ATAD3B | 62 | 3 |
| DNA-dependent protein kinase catalytic subunit GN=PRKDC | 62 | 2 |
| NADH dehydrogenase [ubiquinone] iron-sulfur protein 3, mitochondrial GN=NDUFS3 | 59 | 1 |
| 28S ribosomal protein S7, mitochondrial GN=MRPS7 | 57 | 1 |
| Keratin, type II cytoskeletal 78 GN=KRT78 | 55 | 1 |
| Probable ATP-dependent RNA helicase DDX41 GN=DDX41 | 55 | 2 |
| Immunoglobulin heavy variable 3-64D GN=IGHV3-64D | 53 | 1 |
| Putative RNA-binding protein Luc7-like 2 GN=LUC7L2 | 53 | 2 |
| Pinin GN=PNN | 53 | 2 |
| Insulin-like growth factor 2 mRNA-binding protein 1 GN=IGF2BP1 | 52 | 3 |
| Proteasome subunit alpha type-6 GN=PSMA6 | 51 | 1 |
| Rabankyrin-5 GN=ANKFY1 | 50 | 2 |
| Poly(rC)-binding protein 1 GN=PCBP1 | 49 | 1 |
| Interleukin enhancer-binding factor 3 GN=ILF3 | 49 | 2 |
| Splicing factor U2AF 65 kDa subunit GN=U2AF2 | 49 | 1 |
| Far upstream element-binding protein 2 GN=KHSRP | 46 | 1 |
| Histone H1.1 GN=H1-1 | 46 | 1 |
| Stress-induced-phosphoprotein 1 GN=STIP1 | 45 | 1 |
| Prelamin-A/C GN=LMNA | 45 | 1 |
| U2 small nuclear ribonucleoprotein A~ GN=SNRPA1 | 43 | 2 |
| Isoleucine--tRNA ligase, cytoplasmic GN=IARS1 | 42 | 1 |
| Putative annexin A2-like protein GN=ANXA2P2 | 40 | 1 |
| BTB/POZ domain-containing protein 9 GN=BTBD9 | 39 | 2 |
| U5 small nuclear ribonucleoprotein 200 kDa helicase GN=SNRNP200 | 39 | 1 |
| Coiled-coil domain-containing protein 9 GN=CCDC9 | 38 | 1 |
| Copine-9 GN=CPNE9 | 37 | 1 |
| Transketolase GN=TKT | 37 | 1 |
| U2 small nuclear ribonucleoprotein B~~ GN=SNRPB2 | 37 | 1 |
| Protein NipSnap homolog 2 GN=NIPSNAP2 | 37 | 1 |
| Serine/threonine-protein phosphatase 1 regulatory subunit 10 GN=PPP1R10 | 36 | 1 |
| Pre-mRNA-splicing factor ATP-dependent RNA helicase DHX15 GN=DHX15 | 35 | 2 |
| WD repeat-containing protein 27 GN=WDR27 | 34 | 1 |
| Proteasome subunit alpha type-4 GN=PSMA4 | 33 | 1 |
| Dolichyl-diphosphooligosaccharide--protein glycosyltransferase subunit 2 GN=RPN2 | 33 | 1 |
| Phosphoglycerate kinase 1 GN=PGK1 | 33 | 1 |
| Extracellular matrix protein FRAS1 GN=FRAS1 | 32 | 1 |
| Heterogeneous nuclear ribonucleoprotein U-like protein 1 GN=HNRNPUL1 | 31 | 1 |
| Proteasome subunit alpha type-1 GN=PSMA1 | 31 | 1 |
| Cyclin-dependent kinase 20 GN=CDK20 | 31 | 1 |
| Inorganic pyrophosphatase GN=PPA1 | 31 | 1 |
| Serotransferrin GN=TF | 31 | 1 |
| Inter-alpha-trypsin inhibitor heavy chain H3 GN=ITIH3 | 30 | 1 |
| 40S ribosomal protein S25 GN=RPS25 | 29 | 1 |
| Serpin B12 GN=SERPINB12 | 29 | 1 |
| Bcl-2-associated transcription factor 1 GN=BCLAF1 | 28 | 1 |
| Serine/arginine-rich splicing factor 6 GN=SRSF6 | 27 | 1 |
| CTP synthase 1 GN=CTPS1 | 27 | 1 |
| Olfactory receptor 10X1GN=OR10X1 | 27 | 1 |
| Cytochrome P450 4F3 GN=CYP4F3 | 27 | 1 |
| YLP motif-containing protein 1 GN=YLPM1 | 26 | 1 |
| Protein S100-A9 GN=S100A9 | 26 | 1 |
| Heterogeneous nuclear ribonucleoprotein H GN=HNRNPH1 | 26 | 1 |
| Ubiquitin-associated protein 2-like GN=UBAP2L | 26 | 1 |
| Splicing factor 3A subunit 2 GN=SF3A2 | 26 | 1 |
| Coiled-coil domain-containing protein 189 GN=CCDC189 | 26 | 1 |
| Prolactin-inducible protein GN=PIP | 25 | 1 |
| Dual oxidase 2 GN=DUOX2 PE=1 SV=2 | 25 | 1 |
| Desmocollin-1 OS=Homo sapiens OX=9606 GN=DSC1 | 25 | 1 |
| Serine/threonine-protein kinase ULK4 GN=ULK4 | 25 | 1 |
| Mitogen-activated protein kinase 1 GN=MAPK1 | 24 | 1 |
| Aflatoxin B1 aldehyde reductase member 4 GN=AKR7L | 24 | 1 |
| Splicing factor 3B subunit 2 GN=SF3B2 | 24 | 1 |
| Ras GTPase-activating protein-binding protein 1 GN=G3BP1 | 24 | 1 |
| Hemopexin GN=HPX | 23 | 1 |
| Dynein heavy chain 12, axonemal GN=DNAH12 | 23 | 1 |
| Peptidyl-prolyl cis-trans isomerase FKBP3 GN=FKBP3 | 22 | 1 |
| 5~-3~ exonuclease PLD4 GN=PLD4 | 18 | 1 |
| DNA-directed RNA polymerase I subunit RPA1 GN=POLR1A | 17 | 1 |
| Telomeric repeat-binding factor 2 GN=TERF2 | 14 | 1 |
