# Supplementary figures and images for: TIPE3 represses head and neck squamous cell carcinoma progression via triggering PGAM5 mediated mitochondria dysfunction
Source: Cell Death Dis. 2023 Apr 6;14(4):251. doi: 10.1038/s41419-023-05775-3 (PMC10079926; doi:10.1038/s41419-023-05775-3)

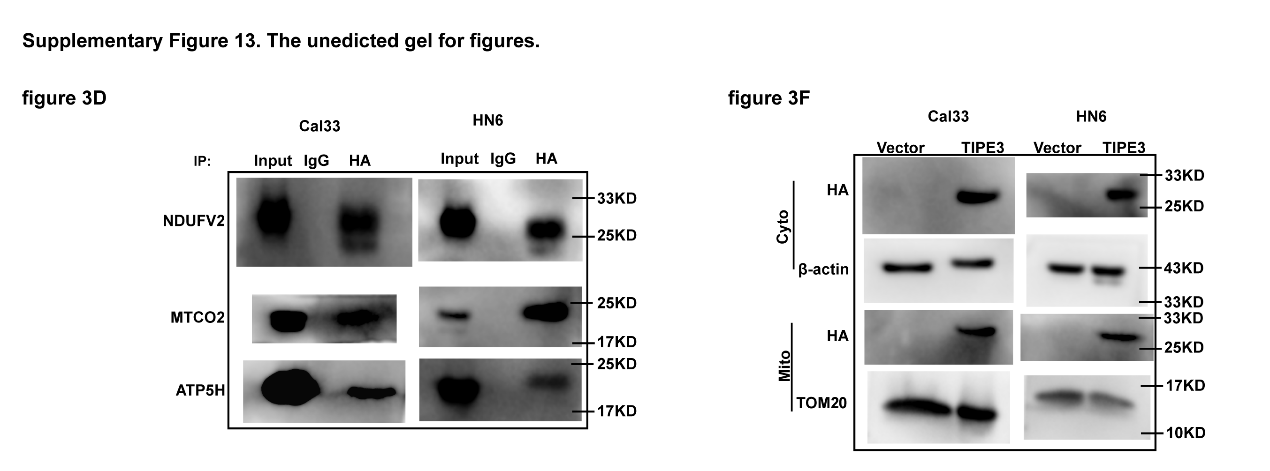


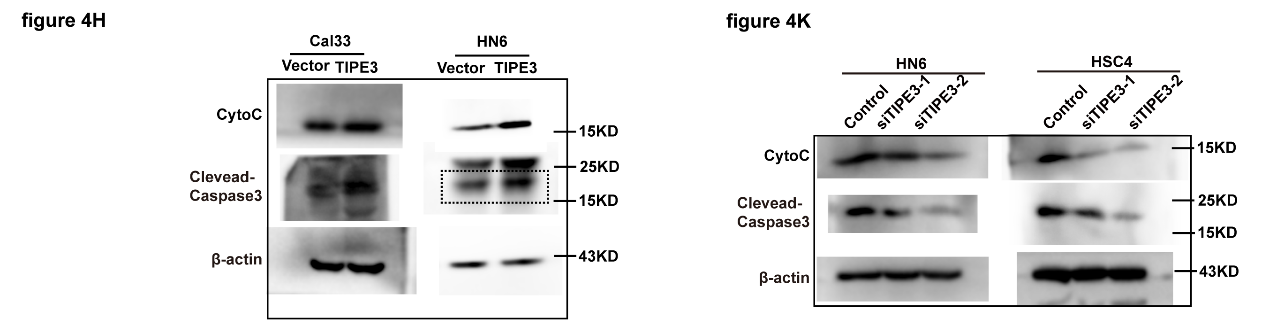


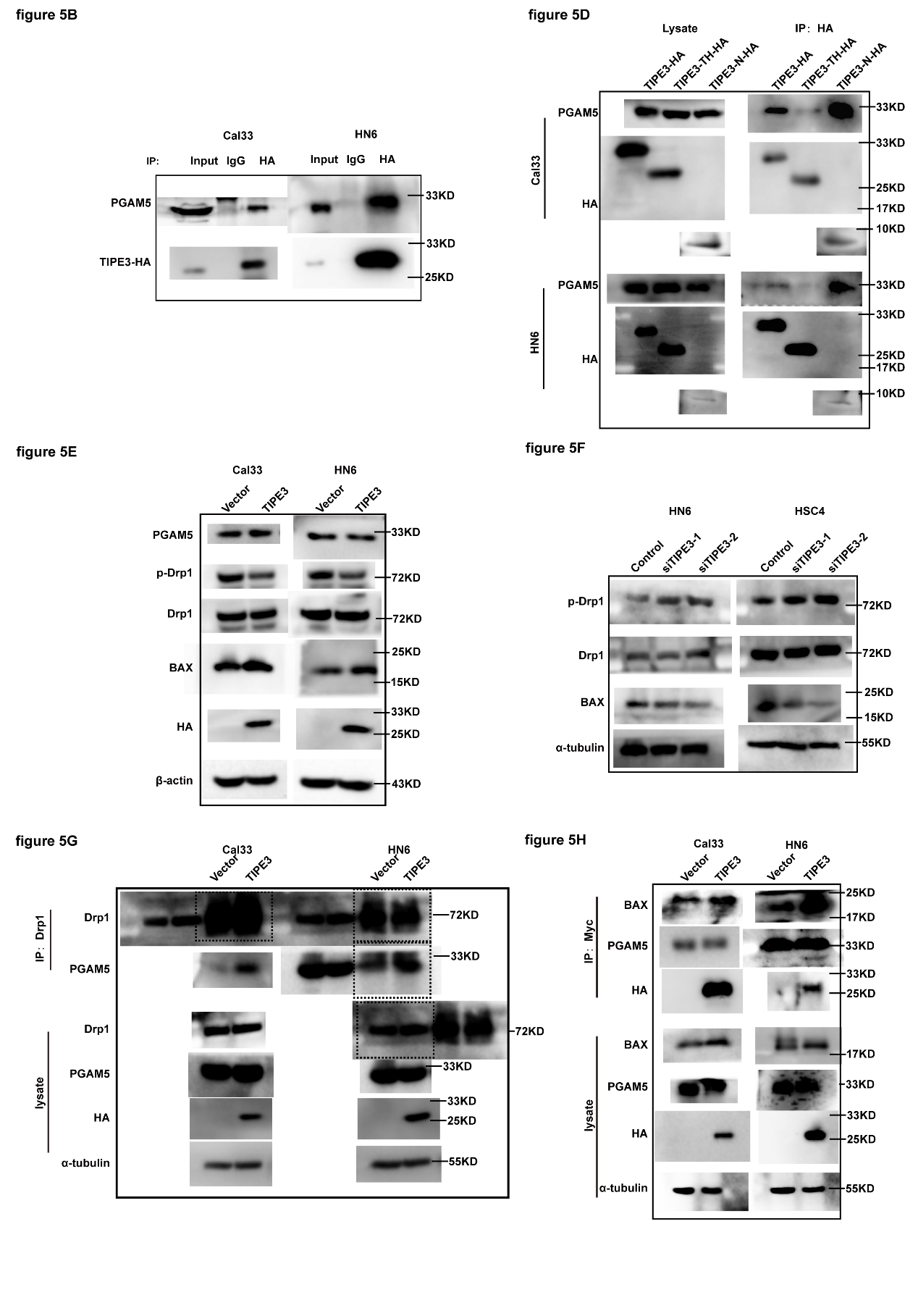

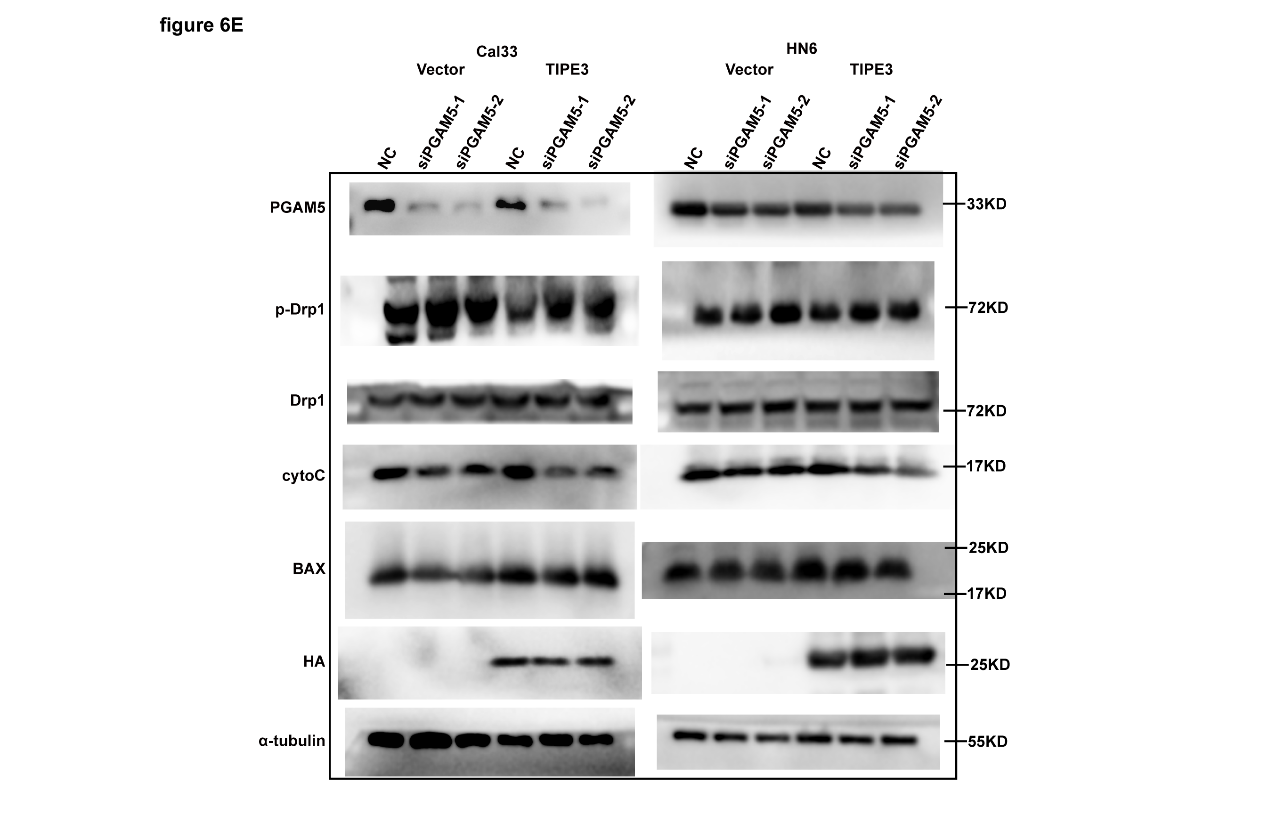


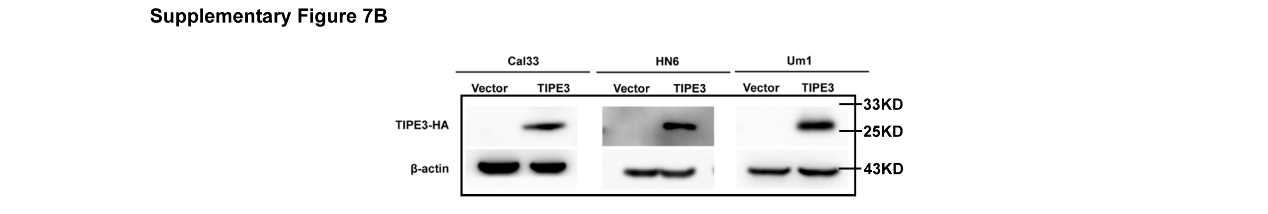


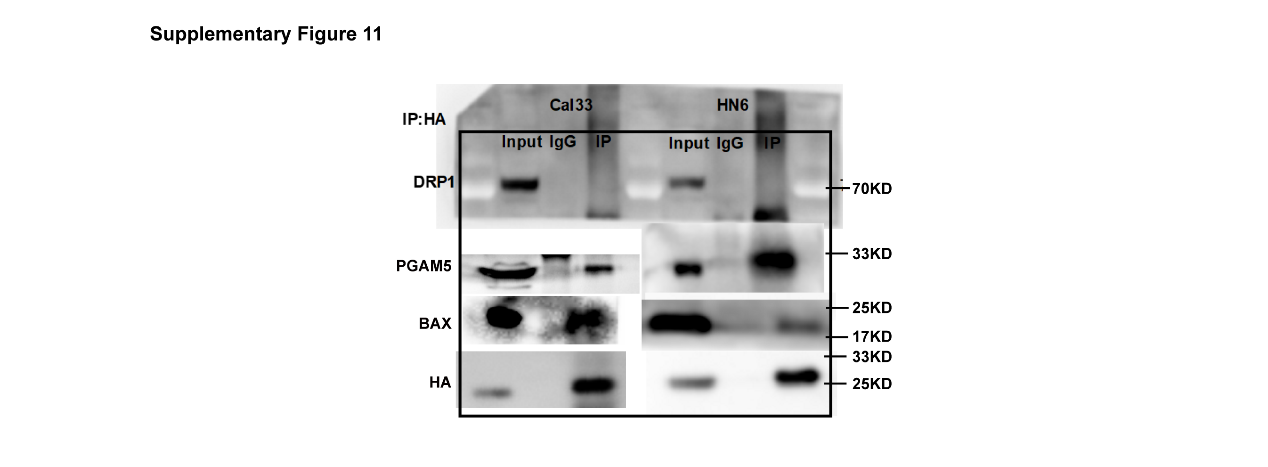


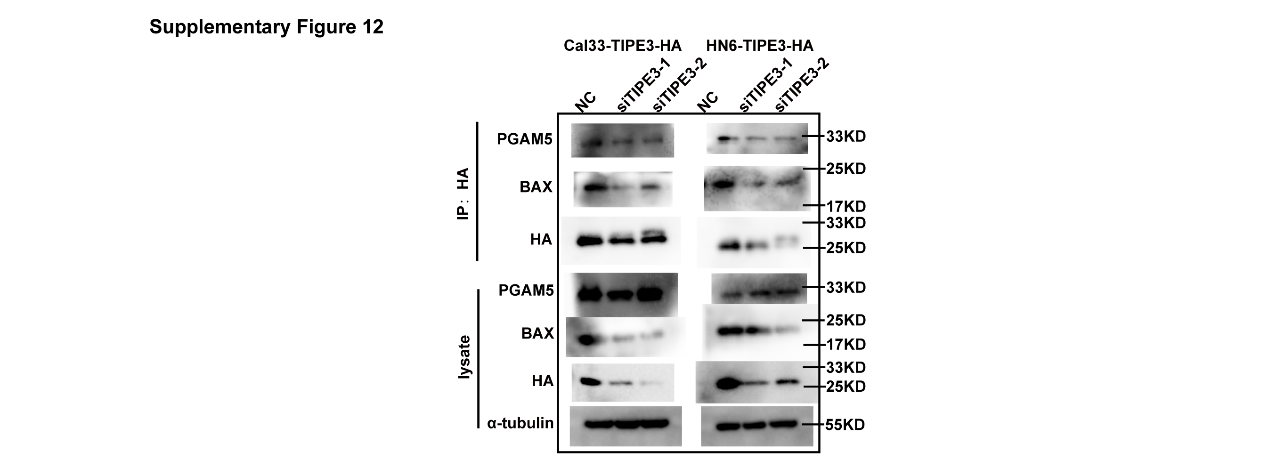

Supplement: Supplementary file 3 — Unedited gel for figures [file 41419_2023_5775_MOESM3_ESM.docx]
